# Subgenome-aware analyses suggest a reticulate allopolyploidization origin in three *Papaver* genomes

**DOI:** 10.1038/s41467-023-37939-2

**Published:** 2023-04-19

**Authors:** Ren-Gang Zhang, Chaoxia Lu, Guang-Yuan Li, Jie Lv, Longxin Wang, Zhao-Xuan Wang, Zhe Chen, Dan Liu, Ye Zhao, Tian-Le Shi, Wei Zhang, Zhao-Hui Tang, Jian-Feng Mao, Yong-Peng Ma, Kai-Hua Jia, Wei Zhao

**Affiliations:** 1 Yunnan Key Laboratory for Integrative Conservation of Plant Species with Extremely Small Populations / Key Laboratory for Plant Diversity and Biogeography of East Asia, Kunming Institute of Botany, Chinese Academy of Sciences, Kunming, 650201 Yunnan China; 2 University of the Chinese Academy of Sciences, 100049 Beijing, China; 3 Key Laboratory of Crop Genetic Improvement & Ecology and Physiology, Institute of Crop Germplasm Resources, Shandong Academy of Agricultural Sciences, Jinan, 250100 Shandong China; 4 Department of Bioinformatics, Ori (Shandong) Gene Science and Technology Co., Ltd., Weifang, 261322 Shandong China; 5 College of Life Science and Technology, Beijing University of Chemical Technology, 100029 Beijing, China; 6 School of Biological Science and Technology, University of Jinan, Jinan, 250022 Shandong China; 7 Shijiazhuang People’s Medical College, Shijiazhuang, 050091 Hebei China; 8 InvoGenomics Biotechnology Co., Ltd., Jinan, 250109 Shandong China; 9 Shandong Provincial Center of Forest and Grass Germplasm Resources, Jinan, 250102 Shandong China; 10 National Engineering Research Center of Tree Breeding and Ecological Restoration, State Key Laboratory of Tree Genetics and Breeding, Key Laboratory of Genetics and Breeding in Forest Trees and Ornamental Plants, Ministry of Education, College of Biological Sciences and Technology, Beijing Forestry University, 100083 Beijing, China; 11 Department of Ecology and Environmental Science, Umeå University, SE-901 87 Umeå, Sweden

**Keywords:** Plant evolution, Adaptive radiation, Evolutionary genetics, Genome evolution

**arising from** X. Yang et al. *Nature Communications* 10.1038/s41467-021-26330-8 (2021)

Hybridization and polyploidization are important driving forces in angiosperm evolution, resulting in novel phenotypes capable of prompting ecological diversification and invasion of new niches^1^. The genus *Papaver* (Papaveraceae) contains many taxa used in the pharmaceutical and culinary industries and as ornamental plants^2^. Yang et al. assembled de novo two chromosome-level genomes of *P. rhoeas* (common poppy, 2n = 14) and *P. setigerum* (Troy poppy, 2n = 44), and improved the *P. somniferum* genome assembly (opium poppy, 2n = 22)^3^. These high-quality, chromosome-scale genome assemblies represent a valuable resource for studying the early evolutionary history of eudicots and the evolution of morphinan biosynthesis. Based on synteny and phylogenomic analyses, the authors identified two rounds of whole-genome duplication (WGD), one in the ancestor to *P. setigerum* and *P. somniferum* (WGD-1) at ~7.2 million years ago (MYA), and one lineage-specific WGD-2 in *P. setigerum* at ~4.0 MYA. In the absence of effective subgenome-phasing techniques, they proposed complex models to explain the extensive genome reorganization and gene family evolution built upon the duplication of the genome itself (their Figs. 2–4 and Supplementary Figs 27–34). Leveraging the recent developments in subgenome-phasing method published by Jia et al.^4^, we propose an alternative model, i.e., reticulate allopolyploidization, to account for the evolution and the genomic diversity of these three *Papaver* species. Our hypothesis is supported by the following lines of evidence:

1. We extracted 4,791 anchor genes from the inter-genomic syntenic blocks at a ratio of 1:2:4 in *P. rhoeas*, *P. somniferum* and *P. setigerum* using OrthoFinder v2.3.1^5^ and MCScanX^6^ (Supplementary Figs. 1A, 2–3). According to the WGD model proposed by Yang et al.^3^, *P. setigerum* should have two sister-pairs of homoeologous subgenomes appearing as sisters to the subgenomes of *P. somniferum* (Fig. 1A). We inferred the maximum likelihood (ML) trees for each gene and the concatenated sequences of all genes in the same homoeologous chromosome sets (macro-synteny) using IQ-TREE v1.6.12^7^, with *P. rhoeas* as the outgroup. The top six gene tree topologies, supported by 4,231 (88%) of the 4,791 gene trees (Supplementary Fig. 4), show that orthologous gene pairs from *P. somniferum* and *P. setigerum* group together, and are sister to the homoeologous genes from *P. setigerum*. None of the topologies comprising at least 50 gene trees (Supplementary Fig. 4) agree with the WGD model shown in Fig. 1A, and most gene trees (43% of the 4791) support the hypothesis that *P. somniferum* and *P. setigerum* were derived from a reticulate origin (Supplementary Fig. 4; Fig. 1B). In addition, we obtained 15 groups of concatenated gene trees (macro-synteny trees) with at least 100 syntenic genes, and the topologies of these macro-synteny trees are identical to the most gene trees (Supplementary Fig. 5), which further supports the model presented in Fig. 1B rather than that in Fig. 1A.

**Fig. 1:**
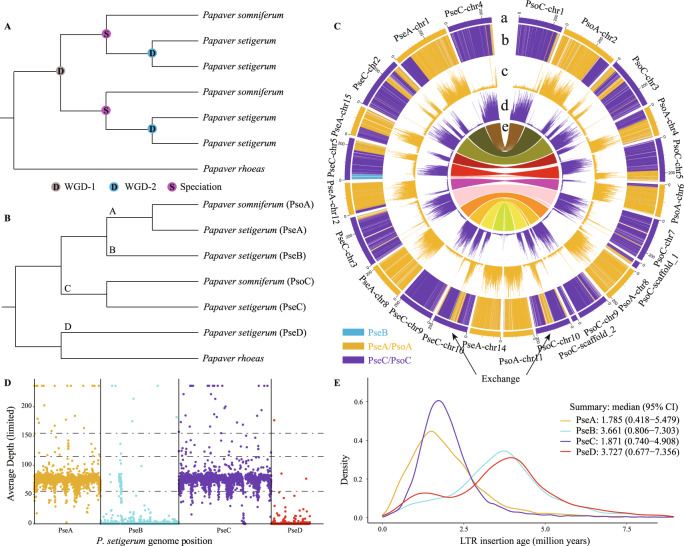
The origin and evolution of the subgenomes in the three studied *Papaver* species. **A** Phylogenomic relationships among the subgenomes assuming the whole-genome duplication (WGD) model of Yang et al.^3^. **B** Tree topology recovered by gene trees, macro-synteny trees, and species/subgenome trees (see Supplementary Figs. 4, 5, 8 for details). The four subgenomes of *P. setigerum* are designated PseA, PseB, PseC and PseD; the two subgenomes of *P. somniferum* are designated PsoA and PsoC, and their ancestors are designated **A**–**D**. **C** Circos plot of subgenome partitions of *P. somniferum* and *P. setigerum* genomes (more details in Supplementary Figs. 6, 7) indicates that PseA and PsoA, and PseC and PsoC share almost all subgenomic exchanges except a segment in PseC-chr5 that shows exchange with PseB. (a) Subgenome assignment of chromosomes based on the *k*-means algorithm. (b) Significant enrichment of subgenome-specific *k*-mers (subgenome partitions). Partitions with the same color as that of a subgenome indicates significant enrichment of *k*-mers specific to that subgenome. The white areas are not significantly enriched. (c–d) Count (absolute) of each subgenome-specific *k*-mer set. (e) Homoeologous blocks between the two species. All statistics (b–d) were computed in sliding windows of 1 Mb. Exchanges between subgenomes, such as that in the middle regions of PseC-chr10 and PsoC-chr10, are inferred from inconsistencies between subgenome assignments calculated using chromosomes (ring a) and windows (rings b–d). **D** The mapping depth of Illumina sequencing reads from *P. somniferum* to *P. setigerum* subgenomes. **E** Insertion times of subgenome-specific LTR-RTs. The 95% confidence intervals (CI) of the insertion times are used to infer the time boundary of divergence to hybridization period.
2. We phased the subgenomes of *P. somniferum* and *P. setigerum* using SubPhaser v1.2^4^ (Supplementary Fig. 1B, C, Supplementary Figs. 6, 7), and extracted orthogroups across 23 species/subgenomes, including two subgenomes of *P. somniferum*, four subgenomes of *P. setigerum*, *P. rhoeas*, and representative lineages of other angiosperms, using OrthoFinder (Supplementary Fig. 8). We then inferred species/subgenome trees using the ML and coalescence-based methods (Supplementary Fig. 8). The topologies of these subgenome trees were consistent with those of the most gene trees (Supplementary Figs. 4, 5), which support the model presented in Fig. 1B. We named the four subgenomes of *P. setigerum* as PseA, PseB, PseC and PseD, and the two subgenomes of *P. somniferum* as PsoA and PsoC according to their phylogenetic relationships (Fig. 1B). Our data suggest that PseA and PsoA, and PseC and PsoC, are derived from separate common ancestors (designated A and C) (Fig. 1B). The A subgenome is sister to PseB, and the combined A/B clade is sister to the C subgenome (Fig. 1B, Supplementary Fig. 8). PseD is sister to *P. rhoeas* and that clade is sister to the combined A + B + C clade (Fig. 1B, Supplementary Fig. 8).
3. We identified exchanges between homoeologous subgenomes in *P. somniferum* and *P. setigerum* using SubPhaser (Supplementary Figs. 6, 7; Supplementary Tables 1, 2). We found that the pattern of exchanges on each chromosome between PsoA and PsoC is almost identical to that between PseA and PseC (except for a single exchange between PseB and PseC; Fig. 1C, Supplementary Figs. 6, 7). We then mapped the Illumina sequencing reads from *P. somniferum* to the *P. setigerum* subgenomes (Supplementary Fig. 1B) using sppIDer^8^. The coverage depth plot showed that almost all the *P. somniferum* reads mapped to PseA and PseC, and very few reads mapped to PseB (i.e. the region exchanged between PseB and PseC) (Fig. 1D). Syntenic dot plots between the subgenomes showed that PsoA and PsoC had greater similarity (lower *Ks*) with PseA and PseC, respectively, but higher *Ks* with PseB and PseD (Supplementary Fig. 2). These results strongly suggest that *P. somniferum* and the two subgenomes PseA and PseC of *P. setigerum* were derived from a common allotetraploid ancestor (designated AC). This suggestion agrees with previous cytological evidence that hybrids between *P. somniferum* (2n = 22) and *P. setigerum* (2n = 44) had around 11 bivalents (mean 10.7II + 11.6I) at metaphase I^9^.
4. There are two possible processes that could lead to the genomic pattern observed in *P. setigerum*: (i) AC hybridized with the ancestors of PseB and PseD separately in a stepwise process; or (ii) the ancestors of PseB and PseD hybridized, forming an allotetraploid (designated BD), then BD hybridized with AC forming the allooctoploid progenitor of *P. setigerum*. To test these two scenarios, we first removed all the potential exchanges between subgenomes of *P. setigerum*, and identified the subgenome-specific long terminal repeat retrotransposons (LTR-RTs) using SubPhaser (Supplementary Fig. 1D). Then we estimated the insertion times of subgenome-specific LTR-RTs in *P. setigerum* to represent the time boundaries from subgenomes differentiation to allohybridization. The estimated PseA- and PseC-specific LTR-RTs insertion times were similar, ranging from ~5 to ~0.5 MYA (95% confidence interval; Fig. 1E). Similarly, the PseB- and PseD-specific LTR-RTs insertion times were also similar (ranging from ~7.3 to ~0.7 MYA) but distinct from those of PseA and PseC (Fig. 1E), suggesting that PseB and PseD were more likely to have been introduced into the *P. setigerum* genome at the same time. Thus, we favored the second scenario, i.e. that the ancestors of PseB and PseD formed an allotetraploid BD, then BD hybridized with AC forming the allooctoploid progenitor of *P. setigerum*.
5. To test whether other potential progenitors were involved in the evolution of these three species, we downloaded all the available sequencing data of *Papaver* species from public databases (see Data Availability for details), and assembled the genes of each species using the HybPiper pipeline^10^. We then extracted 1,474 single-copy genes, and inferred a species tree using ASTRAL-MP v5.14.5^11^. The results suggested that subgenome PseD, *P. rhoeas* and *P. dubium* originated from a common ancestor (Supplementary Fig. 9). Similar to *P. rhoeas*^3^, *P. dubium* showed no evidence of recent WGD (Supplementary Fig. 10), suggesting it could not be a direct tetraploid progenitor (BD) of *P. setigerum*. We did not find closely related species for the subgenomes A, B and C, suggesting either the extinction of related ancestors or a sampling gap in taxon coverage. The tree inferred from the whole chloroplast genomes further suggested that *P. somniferum* was the most likely direct maternal progenitor of *P. setigerum* (Supplementary Fig. 11). Patterns of genome organization in *P. setigerum* and *P. somniferum* suggest that post-polyploidization diploidization is probably still ongoing within the two species as there was no largely biased gene fractionation observed in the subgenomes (Supplementary Fig. 12).

In summary, our comprehensive set of analyses confirmed the two rounds of WGDs previously documented^3^, but we uncovered a reticulate allopolyploidization scenario of evolution in the three studied *Papaver* species (Fig. 2A), involving four ancient diploid genomes (i.e. A, B, C, D) and two tetraploid genomes (i.e. AC and BD). Their most recent common ancestor (MRCA) first diverged into A, B, C, D and *P. rhoeas* at ~4.7–7.3 MYA. B and D then hybridized, resulting in the allotetraploid BD at ~0.91 MYA. The hybridization between A and C occurred ~0.74–0.26 MYA, resulting in the allotetraploid AC, which led to the formation of *P. somniferum* ~0.66 MYA. AC and BD hybridized, resulting in *P. setigerum* at ~0.44 MYA. Genetic exchange between PseB and PseC occurred later. On-going post-polyploidization diploidization resulted in the genome structure we observe in present-day species. However, accurately reconstruction of the genome rearrangements during the allopolyploidization and re-diploidization history remains a challenge with our current methodologies and would require further investigation. Our revision of the speciation and genome evolution model from Yang et al.^3^ has implications for understanding not only the role of reticulation in *Papaver* diversification, but also the evolution of the morphinan and noscapine biosynthesis pathways. Under our genome evolution model, the *STORR* gene fusion event is therefore most likely to have occurred in the ancestor of A and C, or even earlier in the MRCA of this species complex, and was brought to the genomes of *P. somniferum* and *P. setigerum* via hybridization (Fig. 2, Supplementary Figs. 13–15; detailed explanations in Supplementary Note 1), rather than through a post-WGD-1 fusion-translocation event and then duplication following WGD-2, as proposed by Yang et al.^3^. Our model is consistent with a recent study which shows that the *STORR* gene fusion event occurred only once, taking place between 16.8–24.1 MYA, prior to the speciation of this species complex^12^.

**Fig. 2:**
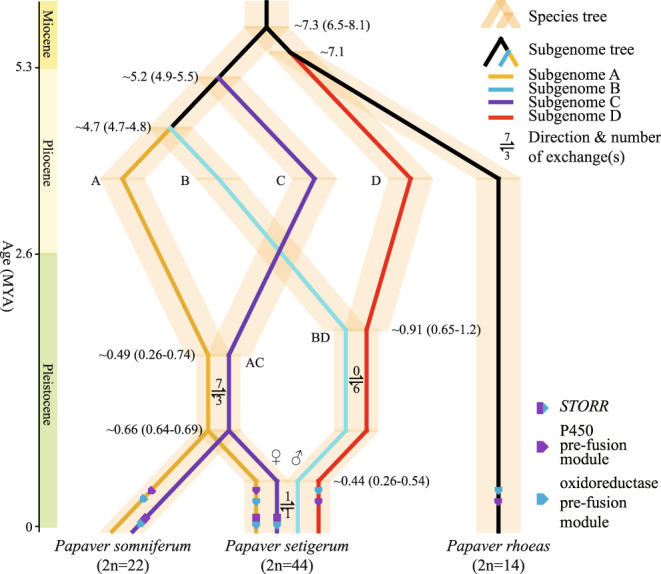
The reticulate allopolyploidization model in the three *Papaver* species and the subgenomic locations of *STORR* and its pre-fusion loci. The times of species/subgenomes divergence and hybridization shown on the topology were based on median *K*s values and the subgenome-specific LTR-RT insertion times, respectively, see Supplementary Tables 3, 4 for details. The currently retained copies of *STORR* and its pre-fusion modules are sorted to subgenomes (see more details in Supplementary Figs. 13–15). Purple and blue denote the cytochrome P450 and oxidoreductase modules, respectively, to correspond with the scheme of Fig. 3a in Yang et al.^3^.

## Methods

### Reconstruction of gene and macro-synteny trees for the three studied *Papaver* species

Syntenic blocks within the three *Papaver* species were identified with OrthoFinder v2.3.1^5^. Orthologous and paralogous relationships, as well as orthogroups, were inferred using the parameters “-M msa -T fasttree” based on proteome sequences from multiple species. The resulting gene pairs were used to call collinear/syntenic blocks using MCScanX (parameters: -a -b 0 -c 0)^6^. For syntenic homologous gene pairs, *K*s was calculated using the ParaAT pipeline^13^ (Supplementary Figs. 2–3). Briefly, the protein sequences of each gene pair were first aligned in MUSCLE v3.8.425^14^, and the alignment was then converted to a codon alignment using PAL2NAL v14^15^. The *K*s was finally calculated using KaKs_Calculator v2.0^16^ with the YN model^17^.

We then extracted 4,791 anchor genes from the inter-genomic syntenic blocks in *P. rhoeas*, *P. somniferum* and *P. setigerum* with a ratio of exactly 1:2:4. To reconstruct the gene trees, the homoeologous gene sequences were aligned with MAFFT v7.481^18^ and trimmed with trimAl v1.2^19^ using a heuristic selection optimized for maximum likelihood (ML) phylogenetic tree reconstruction. Then, the ML tree (Supplementary Fig. 4) was inferred using IQ-TREE v1.6.12^7^ with 1000 bootstraps^20^. The 1:2:4 genes located on the same chromosome set were considered as macro-synteny, and two methods were used to infer the macro-synteny trees (Supplementary Fig. 5): the ML and the coalescence-based method. For the ML method, the gene alignments generated earlier were concatenated, and a tree was reconstructed using IQ-TREE^7^ with 1000 bootstraps^20^. For the coalescence-based method, the gene trees were input into ASTRAL (MP-5.14.5)^11^ to infer the tree based on coalescence.

### Phasing the subgenomes of *P. somniferum* and *P. setigerum*, and inference of species/subgenome trees

We used SubPhaser (parameters: -q 150 -exclude_exchanges)^4^ to phase and partition the subgenomes of *P. somniferum* and *P. setigerum* (Supplementary Figs. 6–7). In brief, chromosomes of a neoallopolyploid were assigned to subgenomes based on differential repetitive *k-*mers that were assumed to have expanded during the period of independent evolution after divergence from the nearest common ancestor and before the hybridization of the parental progenitors (the so-called divergence–hybridization period). A subgenome is considered to be well-phased when it displays distinct patterns of both differential *k*-mers and homoeologous chromosomes, confirming the presence of subgenome-specific features, as expected.

We considered each subgenome as an independent pseudo-species for the subsequent phylogenomic analyses. We additionally collected genomic data from 15 other taxa in the Ranunculales and other angiosperm lineages, as well as RNA-Seq data from *P. bracteatum*, from published papers and public databases (see Data Availability for details). The transcriptome of *P. bracteatum* was first de novo assembled using Trinity v2.6.6^21^ and the coding region of each transcript was annotated using TransDecoder v5.2.0 (https://github.com/TransDecoder/TransDecoder/). Only transcripts with the longest coding region for each gene were retained, and only genes with complete coding regions were used for downstream analyses. We inferred orthogroups from these data using OrthoFinder v2.3.1^5^ as described above. Finally, we inferred the species/subgenome trees (Supplementary Fig. 8) using both ML and coalescence-based methods, as described earlier.

### Identification and validation of exchanges between subgenomes

The identification of exchanges between subgenomes (Supplementary Figs. 6–7, Fig. 1C) was carried out using SubPhaser^4^ in a semi-automated process. SubPhaser assigned each 1 Mb genomic window to subgenomes and flagged windows with enrichments that did not match the subgenome assignments of their chromosome as potential exchanges. These were further checked manually to determine whether they were bona fide exchanges or not. For example, in the middle of C-Pse-chr10 (Supplementary Fig. 6D), subgenome A-specific *k-*mers showed continuous significant enrichments (2nd circle from outer to inner circles), and the abundance of subgenome A-specific *k-*mers was comparable to those on subgenome A chromosomes (4th circle) which contrasted with the other subgenomes (5–7th circles). Based on these observations, we confidently concluded that an exchange had occurred.

After manually checking, we excluded short exchanges with lengths of less than 5 Mb (Supplementary Tables 1–2). As unbalanced exchanges were expected to have syntenic blocks within subgenomes, we validated them through syntenic analyses (Supplementary Figs. 2–3). For example, we observed an exchange where the segment at the 5ʹ end of PseC-15 had been exchanged to the 3ʹ end of PseB-17, resulting in a large syntenic block between PseB-17-3’ and naive PsoB-5-5’ (Supplementary Fig. 2). Due to this imbalance, subgenome PseB now has two copies of this homoeologous segment, leading to a large syntenic block between PseB-17-3’ and PseB-13-3’ where the PseB-naive homoeologous segment is located (Supplementary Fig. 3).

### Comparison of genomic composition of *P. somniferum* and *P. setigerum*

We used the sppIDer^8^ pipeline to confirm the genomic composition of *P. somniferum* and *P. setigerum* (Fig. 1D). This involved mapping short-read sequencing data from *P. somniferum* to the genome of *P. setigerum* to assess the genomic contribution and relative ploidy of each of the subgenomes.

### Identification of potential progenitor species with other *Papaver* species

To identify potential progenitor species, all available sequencing data (i.e. the genome skimming data) from *Papaver* species were downloaded from public databases (see Data Availability for details). The genome skimming data were assembled using the HybPiper pipeline^10^, where the short sequencing reads were mapped to each homologous gene group using BWA-MEM v0.7.17^22^, and assembled with SPAdes v3.13.1^23^. The coding regions were then annotated with exonerate v2.2.0^24^. A total of 1474 single-copy genes were extracted, and a species tree (Supplementary Fig. 9) was inferred using methods described above.

*P. dubium* has the potential to be the tetraploid progenitor (BD) of *P. setigerum*. To explore this possibility further, we downloaded the transcript sequences of *P. dubium* subsp. *lecoqii* from a recent study^12^ and annotated the coding regions using TransDecoder v5.2.0. Next, we inferred orthogroups with OrthoFinder v2.3.1^5^ and calculated *Ks* for both orthologous and paralogous gene pairs using the ParaAT pipeline^13^. Using the *K*s-based method, we inferred potential recent WGD events in *P. dubium* subsp. *lecoqii* (Supplementary Fig. 10).

### Identification of potential maternal parent using the chloroplast tree

To determine the potential maternal parent of *P. somniferum* and *P. setigerum*, we assembled chloroplast genomes from short-read sequencing data of *Papaver* species using GetOrganelle v1.6.2e (parameters: -w 115 -R 13)^25^. The assembled genomes were then annotated using the OGAP pipeline (https://github.com/zhangrengang/ogap). Based on whole plastome sequences of *Papaver* and related taxa, a phylogenomic tree (Supplementary Fig. 11) was inferred using IQ-TREE^7^ with 1000 bootstraps^20^.

### Estimation of divergence and hybridization times

The timing of species/subgenome divergence and hybridization (Supplementary Tables 3–4, Supplementary Figs. 6–7, Fig. 1E) were estimated with two methods: the LTR-based method and the *K*s-based method. Subgenome-specific long terminal repeat retrotransposons (LTR-RTs) are expected to undergo a burst of activity during the divergence–hybridization period. We employed SubPhaser, which uses subgenome-specific LTR-RTs to estimate the upper and lower boundaries of the divergence–hybridization period by applying a symmetric 95% percentile-based confidence interval to the subgenome-specific LTR insertion ages. The analysis excluded any potential exchanged LTR-RTs. Due to the large uncertainty in time estimation using LTR-RTs (particularly for the divergence time)^4^, a traditional *K*s-based method^3^ was also used to estimate the divergence time independently, based on a divergence time of *P. somniferum–P. rhoeas* (7.7 MYA^3^). The estimated times were calculated using the formula **1**:

1 $$T={Ks}/{Ks}(P.{somniferum}{{{{{\rm{\hbox{-}}}}}}}P.{rhoeas})*7.7\,{MYA}$$

assuming an equal substitution rate per year.

### Assignment of subgenome and building gene phylogenies for *STORR*-related loci

Subgenomes for *STORR*-related loci (Supplementary Figs. 13–15, Fig. 2) were determined by their locations on subgenome segments (Supplementary Tables 1–2) using bedtools v2.27.1^26^. Gene trees (Supplementary Figs. 13–14) were reconstructed using IQ-TREE^7^ with 1000 bootstraps^20^.

### Reporting summary

Further information on research design is available in the Nature Portfolio Reporting Summary linked to this article.

## Supplementary information

Supplementary Information

Reporting Summary

## Data Availability

The chloroplast genome sequences assembled in this study have been deposited in the GenBank database under the accession codes OM174280–OM174296. Assemblies of transcriptome and genome skimming data of *Papaver* generated in this study are available at figshare [10.6084/m9.figshare.20323995.v1]. Genome assemblies of *P. rhoeas*, *P. somniferum* and *P. setigerum* were downloaded from the National Genomics Data Center (NGDC) Genome Warehouse (GWH) database under the BioProject accession PRJCA004217. Gene annotations of *P. rhoeas*, *P. somniferum* and *P. setigerum* were downloaded from GitHub [https://github.com/xjtu-omics/Papaver-Genomics/]. Raw genome sequencing reads of *P. rhoeas*, *P. somniferum* and *P. setigerum* were downloaded from the NCBI Sequence Read Archive (SRA) database under the BioProject accession PRJNA720042. Gene annotations of *Macleaya cordata*, *Kingdonia uniflora*, *Tetracentron sinense*, *Coptis chinensis*, and *Prunus persica* were downloaded from the NCBI GenBank/RefSeq databases under the accessions GCA_002174775.1, GCA_014058105.1, GCA_015143295.1, GCA_015680905.1 and GCF_000346465.2, respectively. Gene annotations of *Vitis vinifera* v2.1 [https://phytozome-next.jgi.doe.gov/info/Vvinifera_v2_1] and *Aquilegia coerulea* v3.1 [https://phytozome-next.jgi.doe.gov/info/Acoerulea_v3_1] were taken from the Phytozome database. Gene annotations of *Macadamia integrifolia* were downloaded from the NGDC GWH database under the accession GWHBAUK00000000.1 [https://ngdc.cncb.ac.cn/gwh/Assembly/23196/show]. Gene annotations of *Amborella trichopoda* v6.1 were downloaded from the CoGe database [https://genomevolution.org/coge/GenomeInfo.pl?gid=50948]. Gene annotations of *Trochodendron aralioides* were taken from the GigaDB database [http://gigadb.org/dataset/100657]. Gene annotation of *Coffea canephora* were downloaded from the Coffee Genome Hub [https://coffee-genome-hub.southgreen.fr/node/1/2]. Gene annotations of *Nelumbo nucifera* China Antique v2.0 were downloaded from the *Nelumbo* Genome Database [http://nelumbo.biocloud.net/page/download/download]. Gene annotations of *Eschscholzia californica* v1.0 were from the *Eschscholzia* Genome DataBase [https://drive.google.com/drive/folders/1MIUdVBRBvaIizy75JVI9uh9afd_SYXLo]. Gene annotations of *Aquilegia oxysepala* [10.1038/s41438-020-0328-y] and *Akebia trifoliata* [10.1038/s41438-020-00458-y] were obtained from the corresponding authors. Raw genome skimming sequencing data of 16 *Papaver* species were downloaded from the NCBI SRA database under the BioProject accession PRJEB43865. Raw transcriptome sequencing data of *P. bracteatum* were downloaded from the NCBI SRA database under the BioProject accession PRJEB21674. A transcriptome shotgun assembly of *P. dubium* subsp. *lecoqii* was downloaded from the NCBI GenBank database under the accession GJOS00000000.1. Chloroplast genome sequences of *Papaver* and related taxa were downloaded from the NCBI GenBank/RefSeq databases with accessions MK820043.1, NC_029434.1, NC_037831.1, NC_037832.1, MW411801.1, OK349678.1, MK533647.1, NC_050878.1, NC_056996.1, NC_050877.1, NC_056967.1, NC_039625.1, MK281585.1, NC_039623.1, and NC_029427.1. Source data are provided with this paper.

## Source data

Source Data

## Code availability

The codes used for phasing subgenomes can be found at Github [https://github.com/zhangrengang/SubPhaser]^27^.
