# Supplementary material for: Subgenome-aware analyses suggest a reticulate allopolyploidization origin in three Papaver genomes
Source: Nat Commun. 2023 Apr 19;14:2204. doi: 10.1038/s41467-023-37939-2 (PMC10115784; doi:10.1038/s41467-023-37939-2)
Supplement: Supplementary file 1 — Supplementary Information [file 41467_2023_37939_MOESM1_ESM.pdf]

Subgenome-aware analyses suggest a reticulate allopolyploidization origin in three *Papaver* genomes

Zhang *et al*.

Supplementary Note 1. Revisiting the evolution of *STORR*

Here we use the evolution of *STORR* as an example to explain the significant differences between the two models of the original paper[1] and ours.

Yang et al.[1] discovered that there are two copies of *STORR* in *P. setigerum*, one copy in *P. somniferum* and none in *P. rhoeas*. And they discovered two losses of *STORR* pre-fusion modules in *P. setigerum*, one loss in *P. somniferum* and no losses in *P. rhoeas*, as shown in their Fig. 3a. As *P. setigerum* underwent a lineage-specific WGD (WGD-2), they inferred that the two copies of *STORR* and two losses of *STORR* pre-fusion modules in *P. setigerum* were duplicated from WGD-2, and the MCRA of *P. setigerum* and *P. somniferum* should have only one copy of *STORR* and loss of one *STORR* pre-fusion module (their Fig. 3b). To explain the origin of *STORR*, they proposed a post-WGD-1 fusion-translocation event, in which *STORR* was fused with one copy of the pre-fusion modules that duplicated from WGD-1, and was translocated from its original location to the current *STORR* locus (their Fig. 3b).

However, based on our reticulate allopolyploidization model, we suggest a different evolutionary history of *STORR* as follows.

We assigned subgenomes for the *STORR*-related loci shown in their Fig. 3, and found that the single-copy *STORR* from *P. somniferum* is located in PsoC, and the two copies from *P. setigerum* are located in PseA and PseC separately (our Fig. 2, Supplementary Figs. 13–15). Combined with our allopolyploidization model (Fig. 2, Supplementary Fig. 15), it now is clear that the two copies of *STORR* in *P. setigerum* were inherited from ancestors A and C via hybridization (i.e. both directly from the ancient *P. somniferum*, one progenitor species of *P. setigerum*) (Fig. 2, Supplementary Fig. 15), but were not duplicated from WGD-2. The reason why *STORR* appeared only once in *P. somniferum*, could be attributed to an independent loss of the A-derived *STORR* copy in *P. somniferum* (Supplementary Fig. 15). This hypothesis was supported by the phylogenies of adjacent non-lost genes (Supplementary Fig. 13D–E). Two losses of *STORR* pre-fusion modules occurred in *P. setigerum* (PseC and PseB, respectively) (Fig. 2, Supplementary Figs. 13–15). One loss (PseC) was derived from its progenitor AC, and the other one (PseB) from BD (Fig. 2, Supplementary Fig. 15), rather than from a simple duplication resulted from WGD-2. Our results were also confirmed by the phylogenies of *STORR* with its P450 and oxidoreductase pre-fusion modules (Supplementary Fig. 14).

Based on our model (Fig. 2, Supplementary Fig. 15), we suggested that the *STORR* gene fusion event is therefore likely to have occurred in the ancestor of A and C, or even earlier in the MRCA of this species complex, rather than from a post-WGD-1 fusion-translocation event[1]. Our inference is consistent with a recent study[2] which tracks the gene fusion event of *STORR* in *Papaver* to 16.8–24.1 MYA to be shared with several outgroup *Papaver* species.

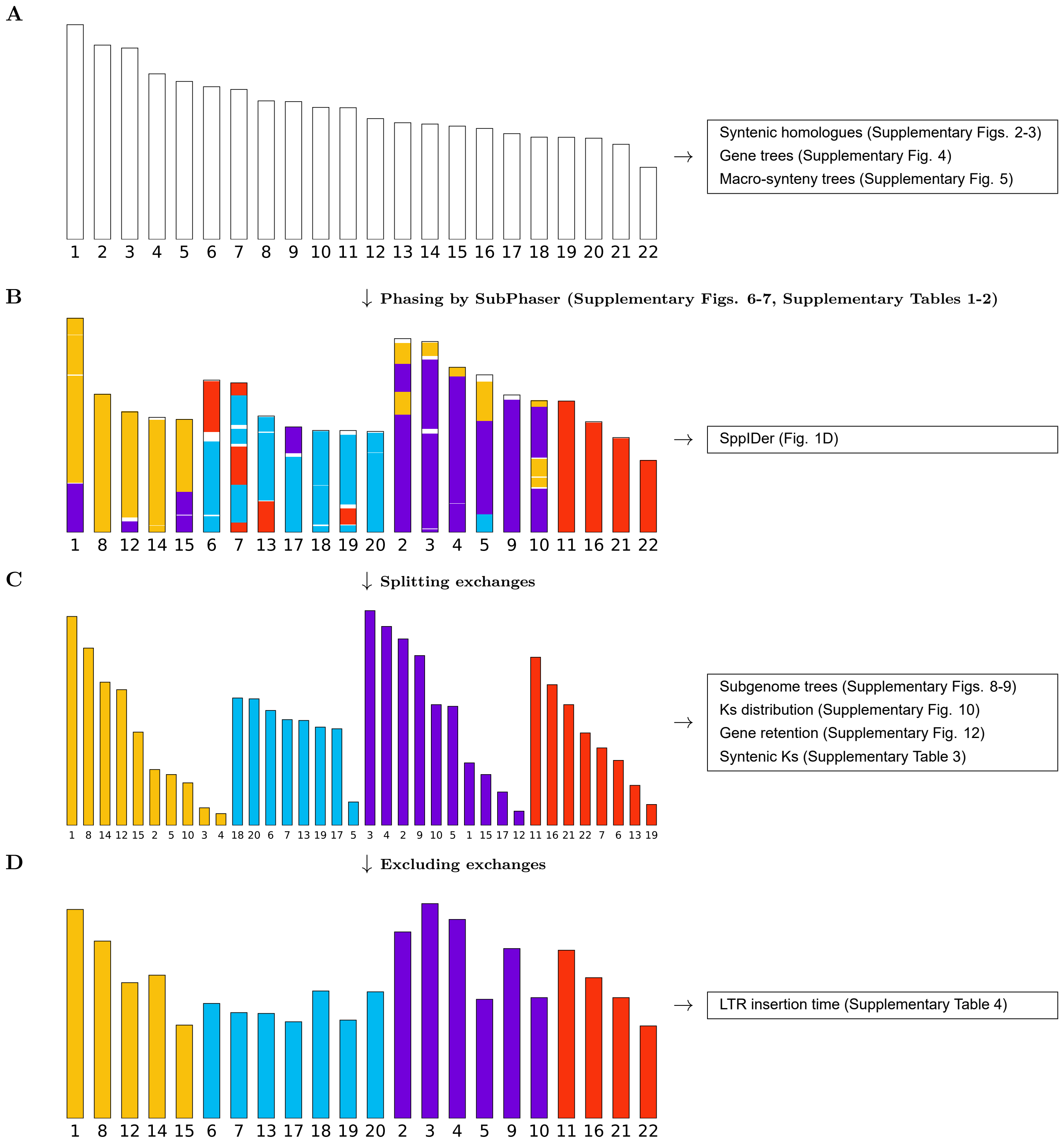

Supplementary Figure 1. Schematic overview of the chromosomal data processing, using *Papaver setigerum* as an example. (**A**) Raw chromosomes. At this stage, we did not have any priors or information regarding subgenomes. We extracted only syntenic homoeologous genes that exactly fitted the relative levels of WGD (i.e. at a ratio of 1:2:4) to construct both the gene trees and the concatenated gene trees (macro-synteny trees) and investigate the relationships between them. (**B**) Subgenome partitioning using SubPhaser. During this stage, we successfully phased the genome into four subgenomes, with each subgenome containing several large-scale exchanges (the minor colored segments). The four colored regions indicate significant enrichments of four subgenome-specific k-mer sets, and white (ambiguous regions) indicates the regions that are either not significantly enriched with specific k-mers or are too short (< 5 Mb). (**C**) Chromosome segments grouped by subgenome. During this stage, exchanges were cut and grouped into corresponding subgenome to study the phylogenomic relationships among subgenomes. Ambiguous regions were directly excluded. (**D**) Chromosome segments with exchanges excluded. During this stage, we aimed to identify subgenome-specific LTRs and estimated their insertion times using SubPhaser. As SubPhaser cannot handle short segments, we simply excluded them to avoid noise.

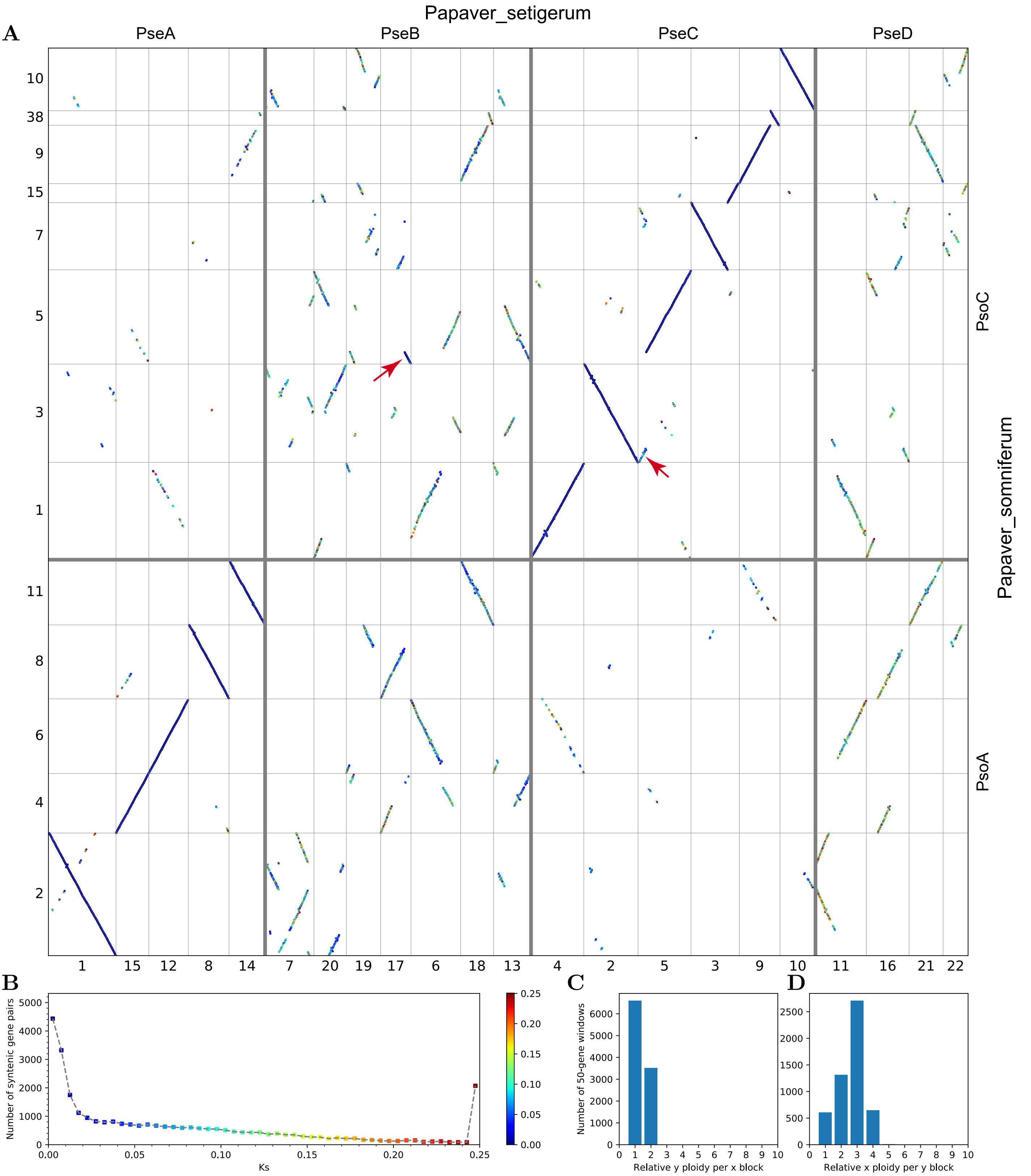

Supplementary Figure 2. Orthologous synteny between *Papaver somniferum* and *Papaver setigerum*. (**A**) Dot plots between the two taxa. A dot indicates a pair of syntenic orthologous genes inferred using OrthoFinder and MCScanX_h sequentially. Dots representing the position of syntenic orthologous gene-pairs were colored according to the synonymous substitution rates (*Ks*) color scale. The smaller the *Ks* value, the higher the homology between the genes. The red arrows indicate unbalanced exchanges that agreed with the exchanges identified by SubPhaser (PseC-chr5 <-> PseB-chr17) (Supplementary Fig. 6). x-axis: chromosomes 1–22 of *Papaver setigerum*; y-axis: chromosomes 1–11 and two large unplaced scaffolds (15 = "scaffold1" and 38 = "scaffold2") of *Papaver somniferum*. (**B**) *Ks* histogram of these syntenic othologous gene pairs (i.e. dots in panel **A**). This panel shares the same *Ks* color map as panel **A**. (**C**–**D**) Syntenic depth from 50-gene windows of x- (**C**) or y- (**D**) axes of panel **A**. Source data are provided as a Source Data file.

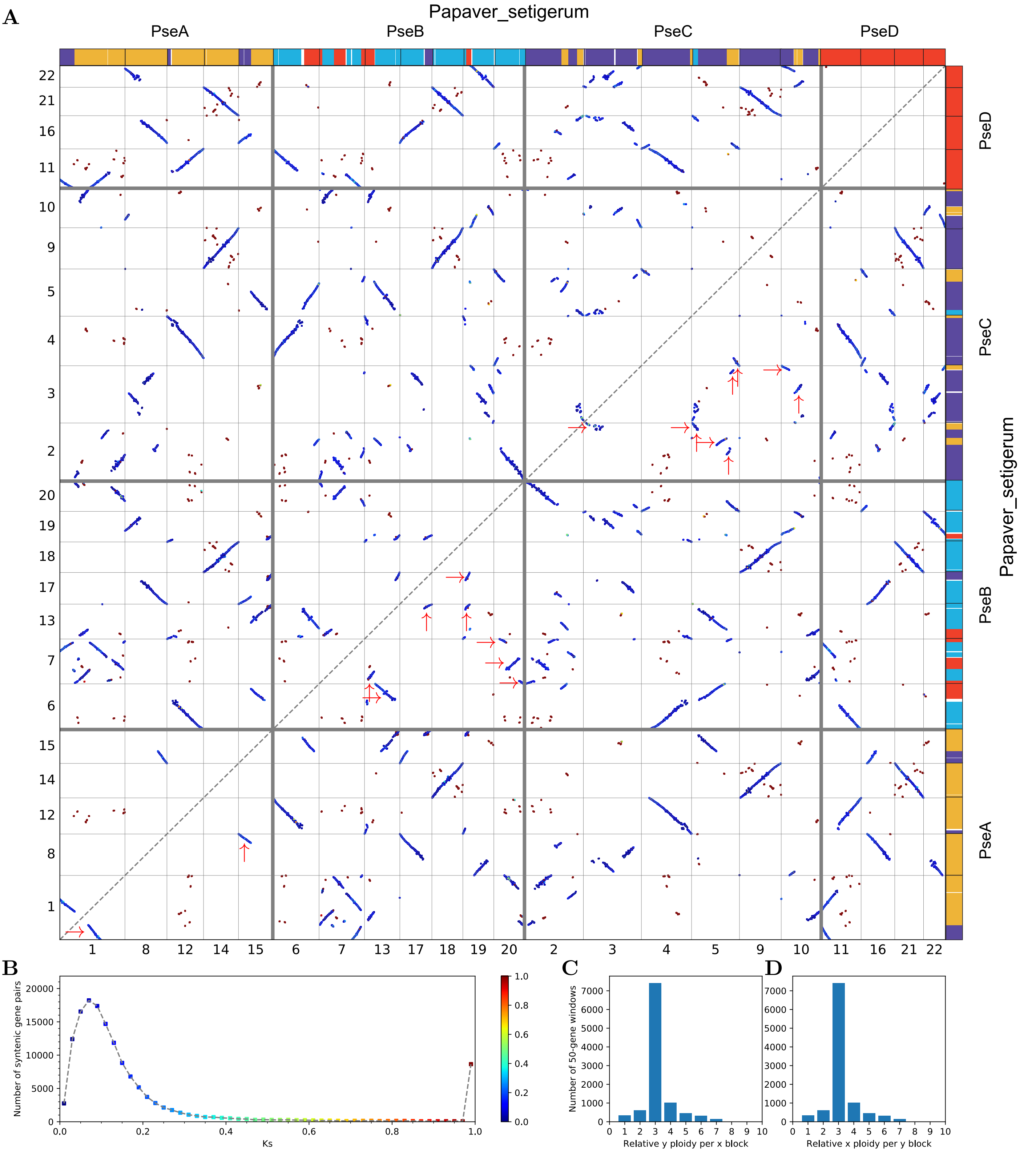

Supplementary Figure 3. Homoeologous synteny within the *Papaver setigerum* genome. (**A**) Dot plots within the genome. A dot indicates a pair of syntenic homoeologous genes inferred using OrthoFinder and MCScanX_h sequentially. The dots are colored according to their synonymous substitution rates (*Ks*). Both x-axis and y-axis: chromosomes 1–22 of *Papaver setigerum*. Note that only the largely intact homoeologous chromosome sets, i.e. (14, 18, 9, 21), (4, 6, 11, 12) and (19, 22, 10), were used to identify differential *k*-mers with SubPhaser. The red arrows indicate unbalanced exchanges (identified by dot plots) that agreed with those exchanges identified by SubPhaser (right and top four-colored panels; Supplementary Fig. 6). The directions (right or up) of arrows indicate with which panel is in agreement. Note that dot plots can not identify balanced homoeologous exchanges or the direction of exchanges. (**B**) *Ks* histogram of these syntenic homoeologous gene pairs (i.e. dots in panel **A**). This panel shares the same *Ks* color map as panel **A**. (**C**–**D**) Syntenic depth from 50-gene windows of x- (**C**) or y- (**D**) axes of panel **A**. Source data are provided as a Source Data file.

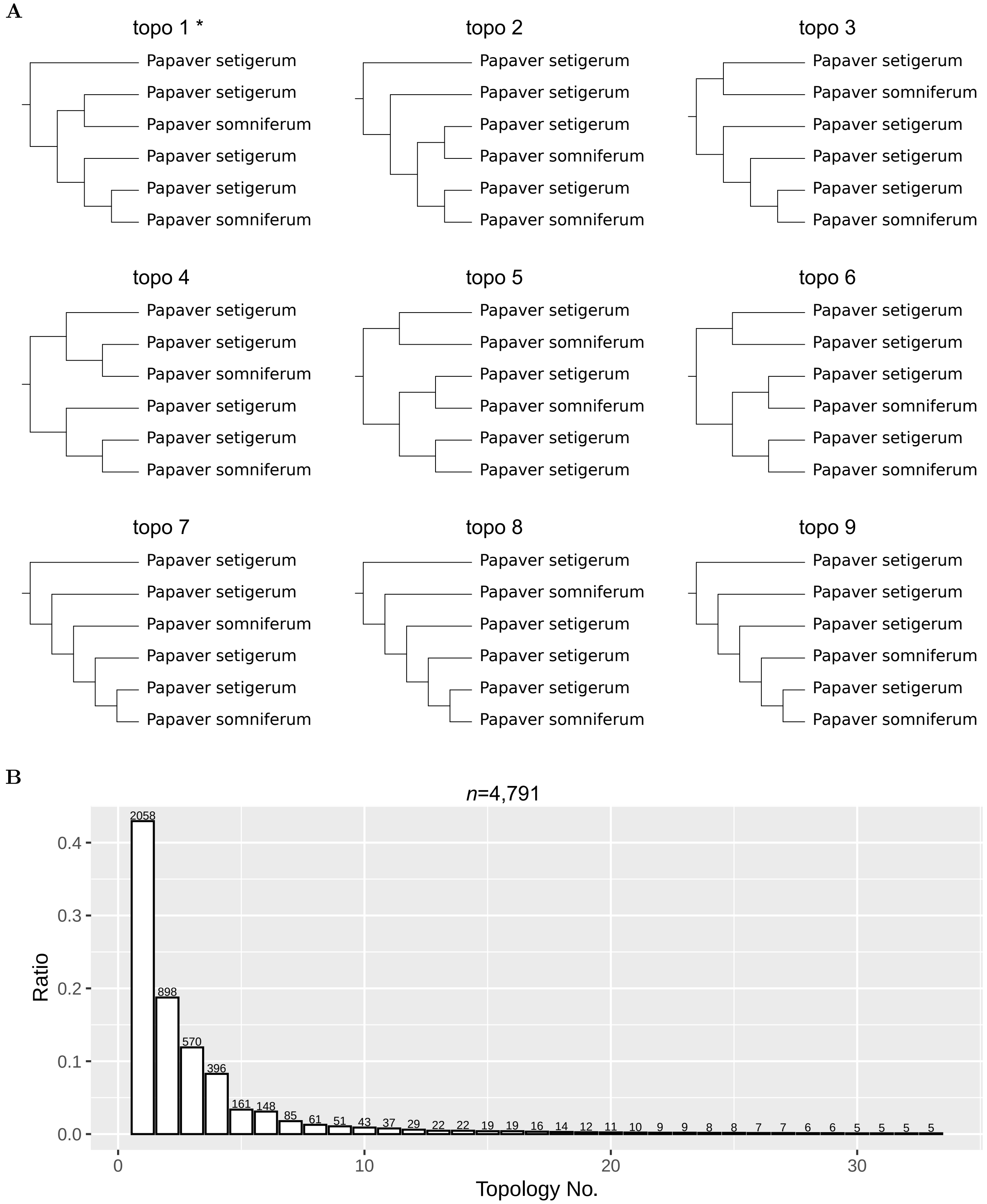

Supplementary Figure 4. Tree topologies of 1:2:4 syntenic orthologous genes. (**A**) Nine topologies with support from at least 50 gene trees. *, topology that suppports our reticulated evolution model. No topology suppports the WGD model of Yang et al.[1]. The trees are rooted with genes from *P. rhoeas* (not shown). The terminal labels sharing a species name indicate the homoeologous genes from that genome. (**B**) Frequency of gene tree topologies. Topologies 1–9 are correspond to the nine topologies in (**A**). The number on the bar is the number of gene trees. Only topologies with at least 5 gene trees are shown. *n*, total number of gene trees. Source data are provided as a Source Data file.

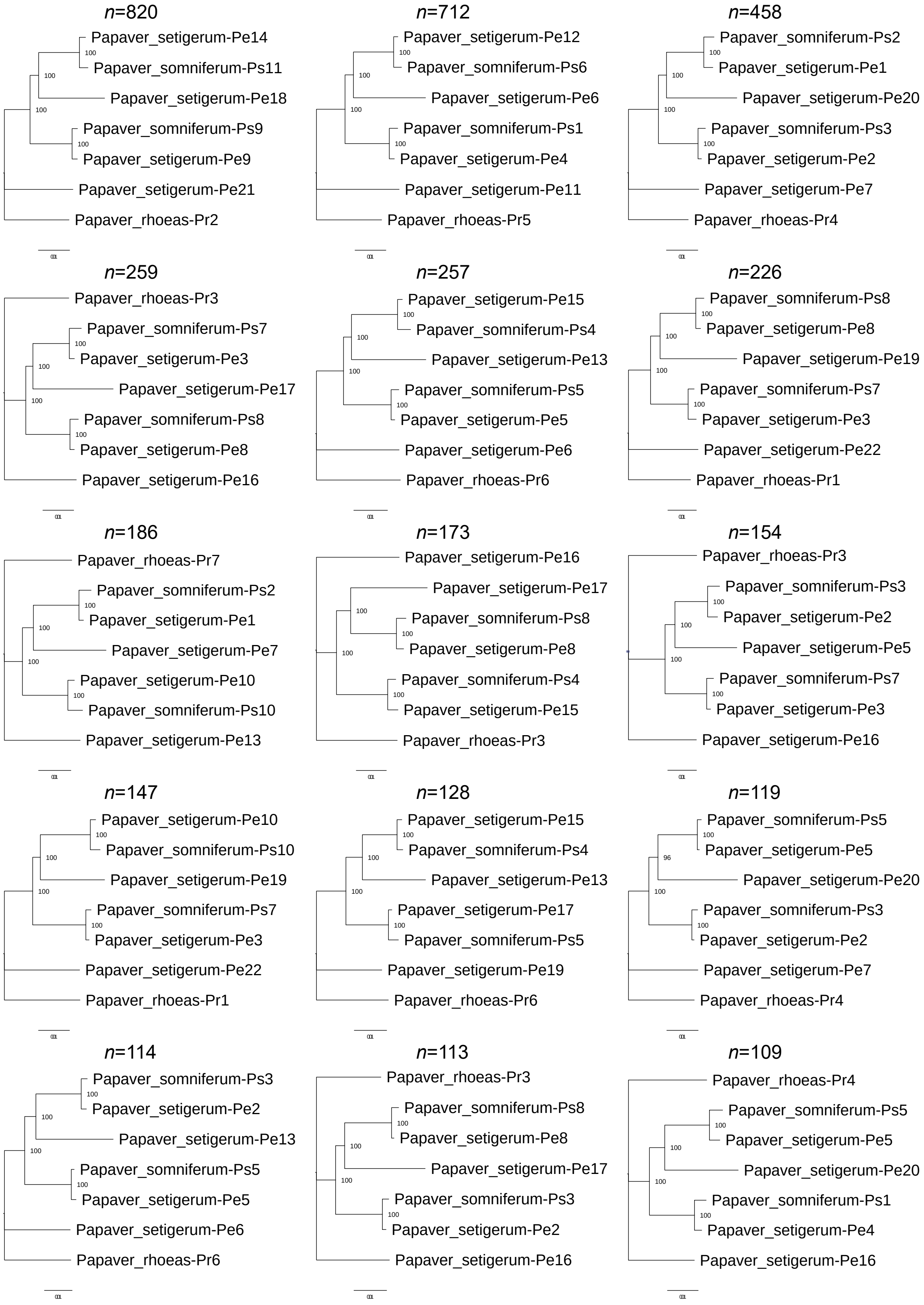

Supplementary Figure 5. Macro-synteny trees inferred from different homoeolgous chromosome sets based on concatenated 1:2:4 syntenic orthologous genes. Syntenic gene alignments were concatenated by their homoeologous chromosome sources and the maximum-likelihood tree was reconstructed using IQ-TREE. Only 15 trees with at least 100 syntenic genes are shown. The trees were rooted with *P. rhoeas*. All trees share the same topology as the topology 1 in Supplementary Fig. 4A. The tip labels on the trees are chromosome numbers. *n*, number of concatenated syntenic genes. Numbers at the nodes represent the bootstrap values. Bar, 0.01 substitutions per site. Source data are provided as a Source Data file.

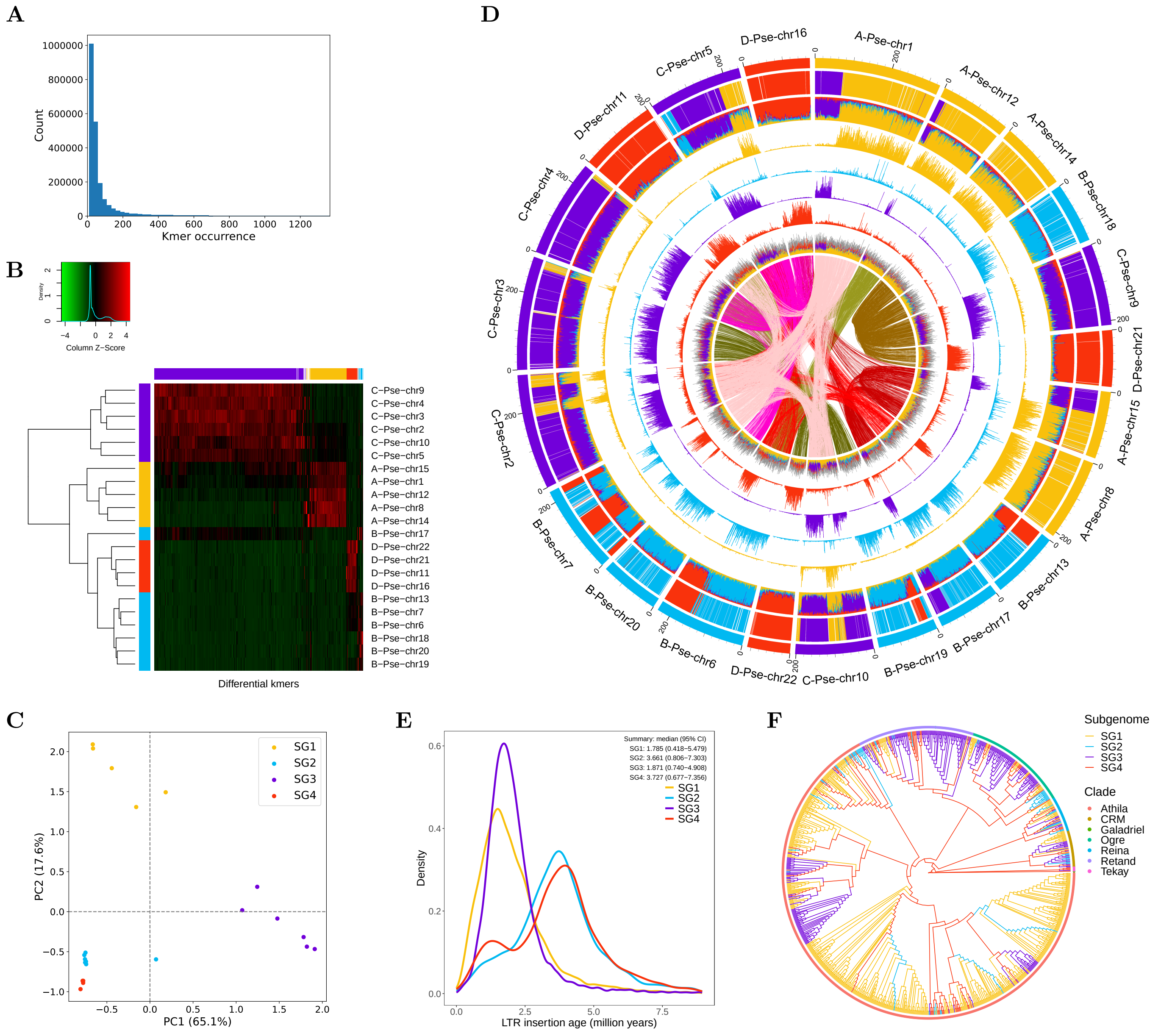

Supplementary Figure 6. Subgenome phasing of *Papaver setigerum* using SubPhaser. (**A**) Frequency distribution of differential 15-mers among the homoeologous chromosomes. (**B**) Unsupervised hierarchical clustering. The horizontal color bar at the top of the axis indicates to which subgenome the k-mer is specific. The vertical color bar on the left of the axis indicates to which subgenome the chromosome is assigned. The heatmap indicates the Z-scaled relative abundance of k-mers; the larger the Z score, the higher the relative abundance of a k-mer. (**C**) Principal component analysis (PCA) of differential 15-mers. (**D**) Chromosomal characteristics. From outer to inner circles (1–9): (1) Subgenome assignments based on k-Means algorithm. (2) Significant enrichment of subgenome-specific k-mers. The same color as the subgenome indicates significant enrichment for those subgenome-specific k-mers. White areas are not significantly enriched. (3) Normalized proportion (relative) of subgenome-specific k-mers. (4–7) Counts (absolute) of each subgenome-specific k-mer set. (8) Density of long terminal repeat retrotransposons (LTR-RTs). If the color is consistent with that of the subgenome, it indicates that LTR-RTs are significantly enriched in those subgenome-specific k-mers. Gray indicates non-specific LTR-RTs. (9) Homoeologous blocks. All statistics (2–8) are computed in sliding windows of 1 Mb. (**E**) Insertion times of subgenome-specific LTR-RTs. The 95% confidence interval (CI) is marked in the upper right corner and was used to predict the insertion time boundaries of LTR-RTs on the subgenome. (**F**) Phylogenetic tree of up to 1,000 *Gypsy* LTR-RTs randomly selected from the subgenome-specific LTR-RTs. The branches are colored by subgenome and the terminal nodes are colored by clade. Clades were classified using TEsorter. (B–F) Colors are consistent for subgenomes. SG1 = PseA, SG2 = PseB, SG3 = PseC, and SG4 = PseD.

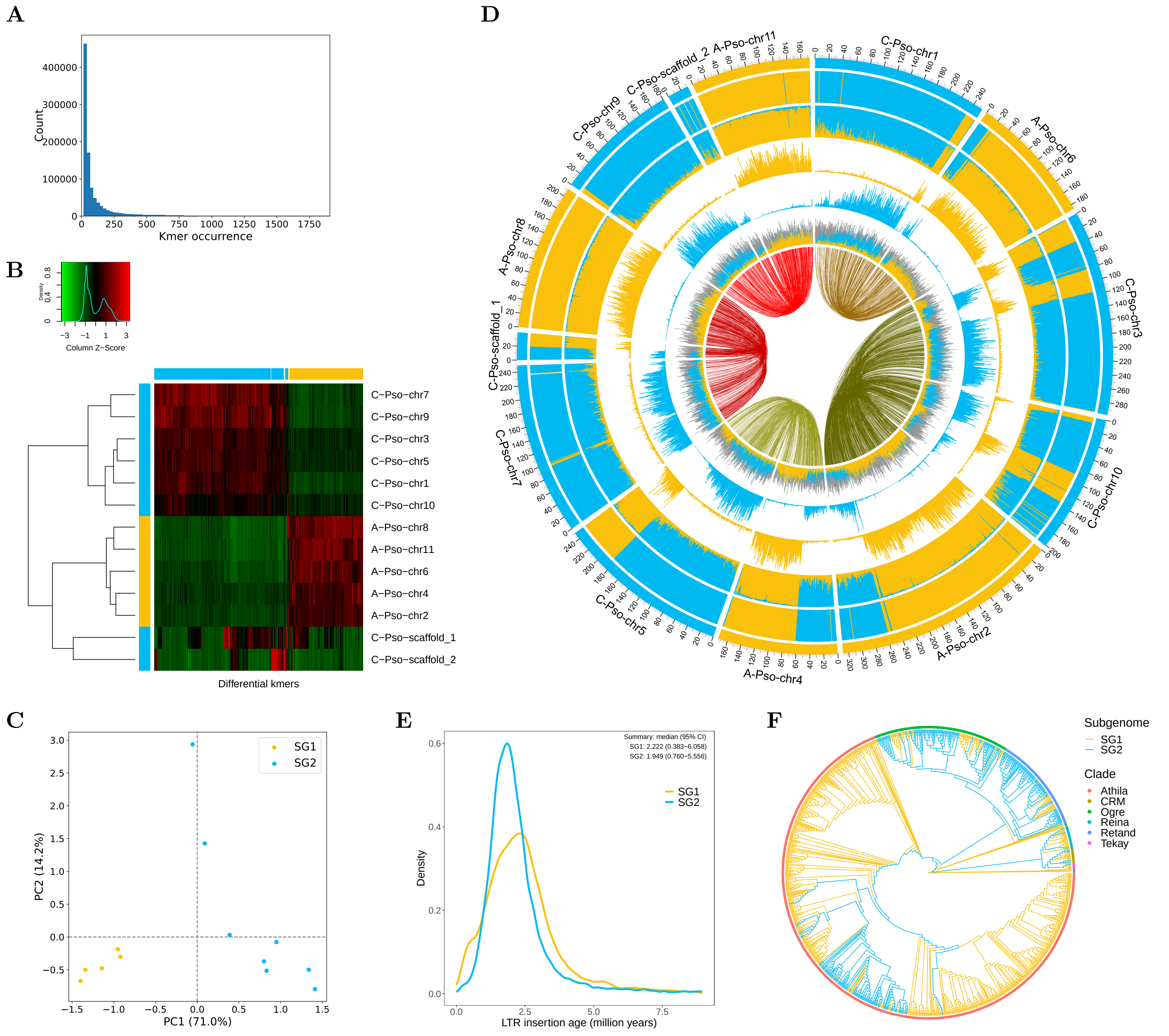

Supplementary Figure 7. Subgenome phasing of *Papaver somniferum* using SubPhaser. (**A**) Frequency distribution of differential 15-mers in the homoeologous chromosomes. (**B**) Unsupervised hierarchical clustering. The horizontal color bar at the top of the axis indicates to which subgenome the k-mer is specific. The vertical color bar on the left of the axis indicates to which subgenome the chromosome is assigned. The heatmap indicates the Z-scaled relative abundance of k-mers; the larger the Z score, the higher the relative abundance of a k-mer. (**C**) Principal component analysis (PCA) of differential 15-mers. (**D**) Chromosomal characteristics. From outer to inner circles (1–7): (1) Subgenome assignments based on a k-Means algorithm. (2) Significant enrichment of subgenome-specific k-mers. The same color as the subgenome indicates significant enrichment for those subgenome-specific k-mers. White areas are not significantly enriched. (3) Normalized proportion (relative) of subgenome-specific k-mers. (4–5) Counts (absolute) for each subgenome-specific k-mer set. (6) Density of long terminal repeat retrotransposons (LTR-RTs). If the color is consistent with that of the subgenome, the LTR-RTs are significantly enriched with those subgenome-specific k-mers. Gray indicates non-specific LTR-RTs. (7) Homoeologous blocks. All statistics (2–6) were computed in sliding windows of 1 Mb. (**E**) Insertion time of subgenome-specific LTR-RTs. The 95% confidence interval (CI) is marked in the upper right-hand corner and was used to predict the insertion time boundaries of LTR-RTs on the subgenome. (**F**) Phylogenetic tree of up to 1,000 *Gypsy* LTR-RTs randomly selected from the subgenome-specific LTR-RTs. The branches are colored by subgenome and the terminal nodes are colored by clades. Clades were classified using TEsorter. (B–F) Colors are consistent for subgenomes. SG1 = PsoA, and SG2 = PsoC.

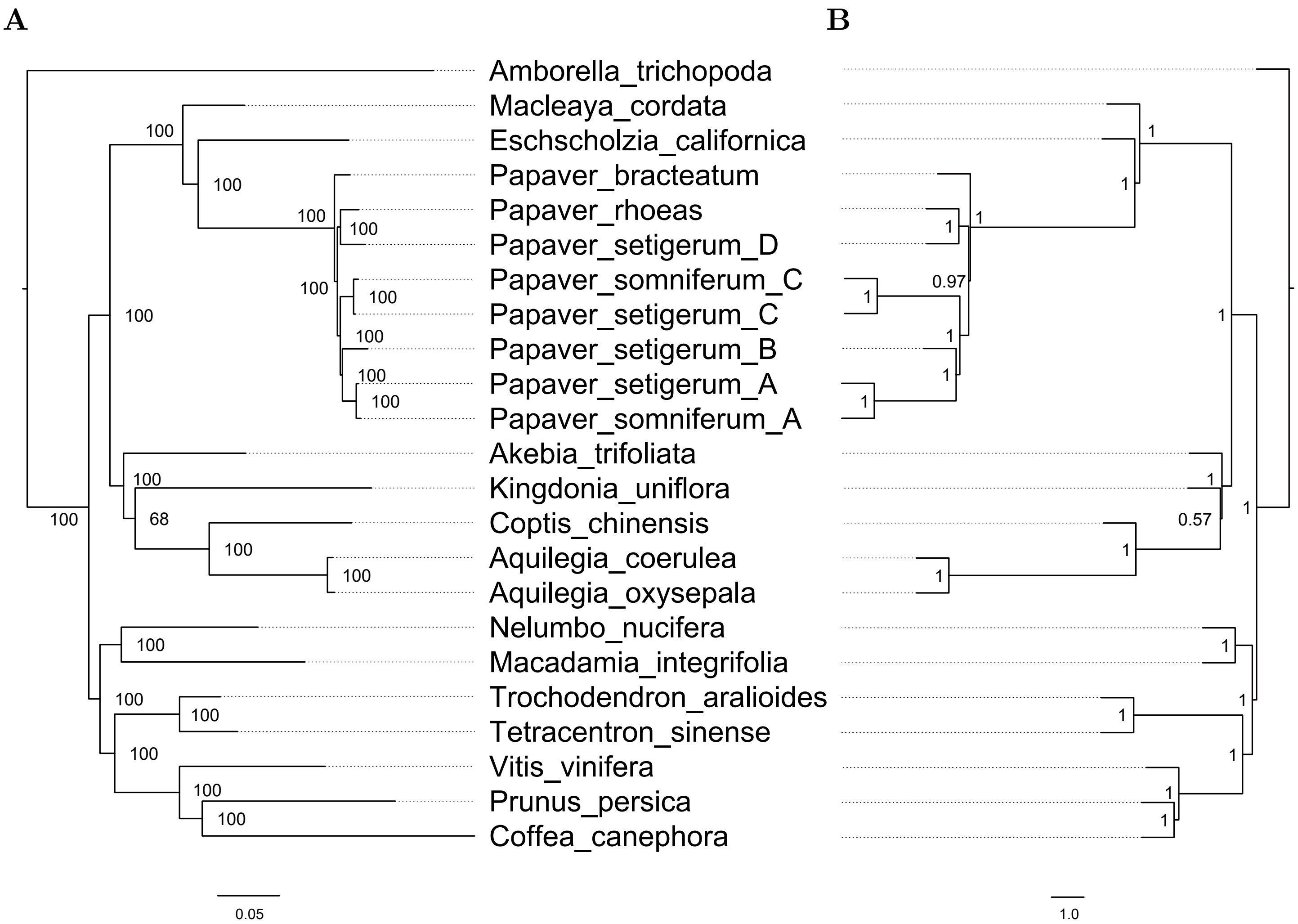

Supplementary Figure 8. Subgenome-aware species trees inferred using (A) maximum likelihood (ML) [IQ-TREE] and (B) coalescent [ASTRAL] approaches. (**A**) The maximum-likelihood tree is based on 908 orthogroups with a minimum of 78.3% of species in any orthogroup having single-copy genes. Numbers at the nodes are the bootstrap values as calculated with IQ-TREE. Bar, 0.05 substitutions per site. (**B**) The coalescent tree was based on 3,874 gene trees with genes in at least 70% taxa being single-copy. Numbers at the nodes represent the local posterior probabilities calculated in ASTRAL. Bar, 1.0 coalescent units. Note the terminal branch length was fixed to 1.0 in the FigTree software for better visualization. The three *Papaver* genomes were download from Yang et al.[1], and *Papaver bracteatum* was assembled from transcriptome data (BioProject accession PRJEB21674). Source data are provided as a Source Data file.

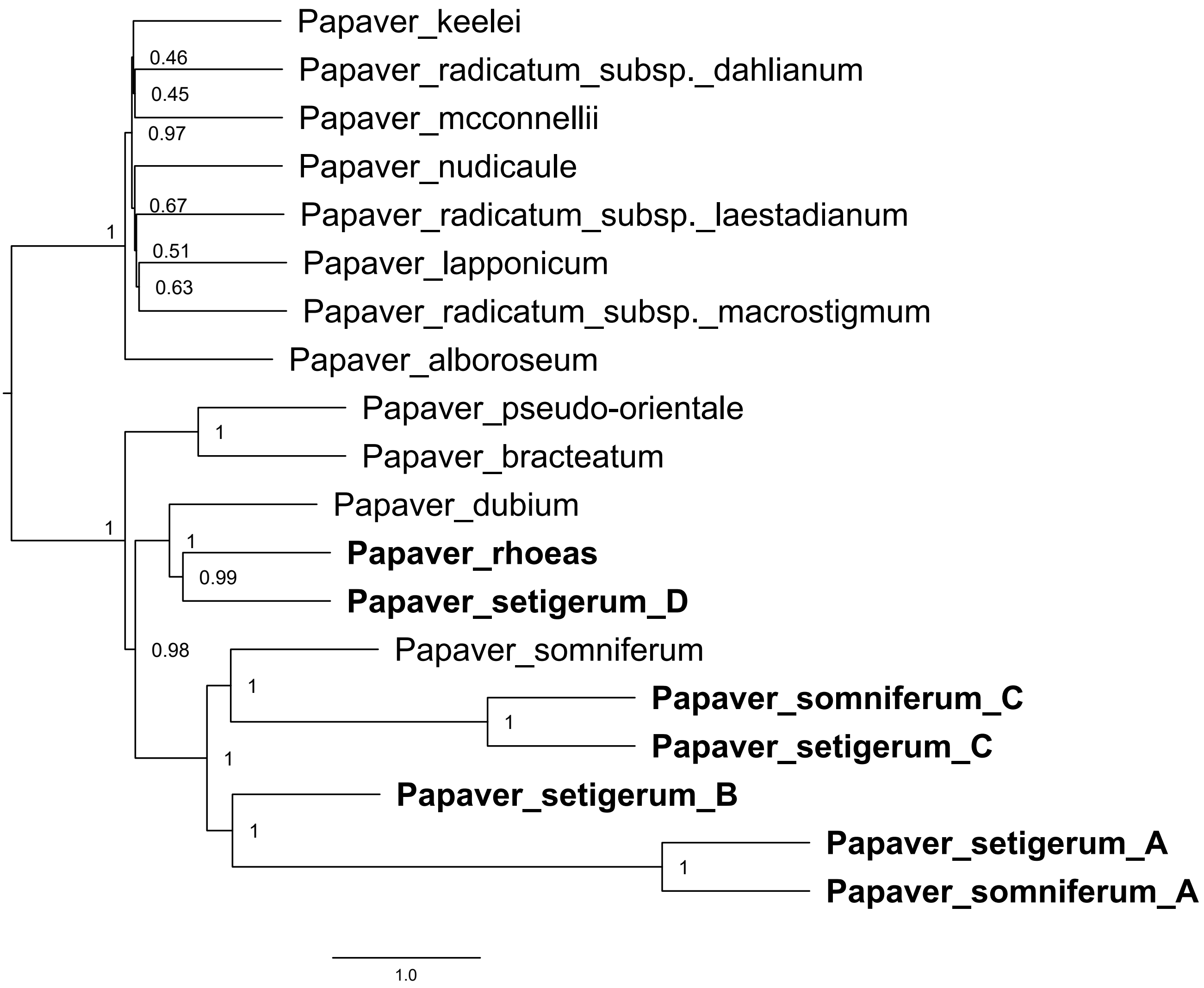

Supplementary Figure 9. Subgenome-aware species trees of *Papaver* genus. Whole genomic data are highlighted in bold. Sequences from other species were assembled from the genome skimming data under the BioProject accession PRJEB43865, except for that of *Papaver bracteatum* which was assembled from transcriptome data (PRJEB21674). The tree was inferred using ASTRAL from 1,474 gene trees. No additional progenitor was found on this tree. Numbers at the nodes represent the local posterior probabilities calculated using ASTRAL. Bar, 1.0 coalescent units. Note the terminal branch length was fixed to 1.0 in the FigTree software for better visualization. Source data are provided as a Source Data file.

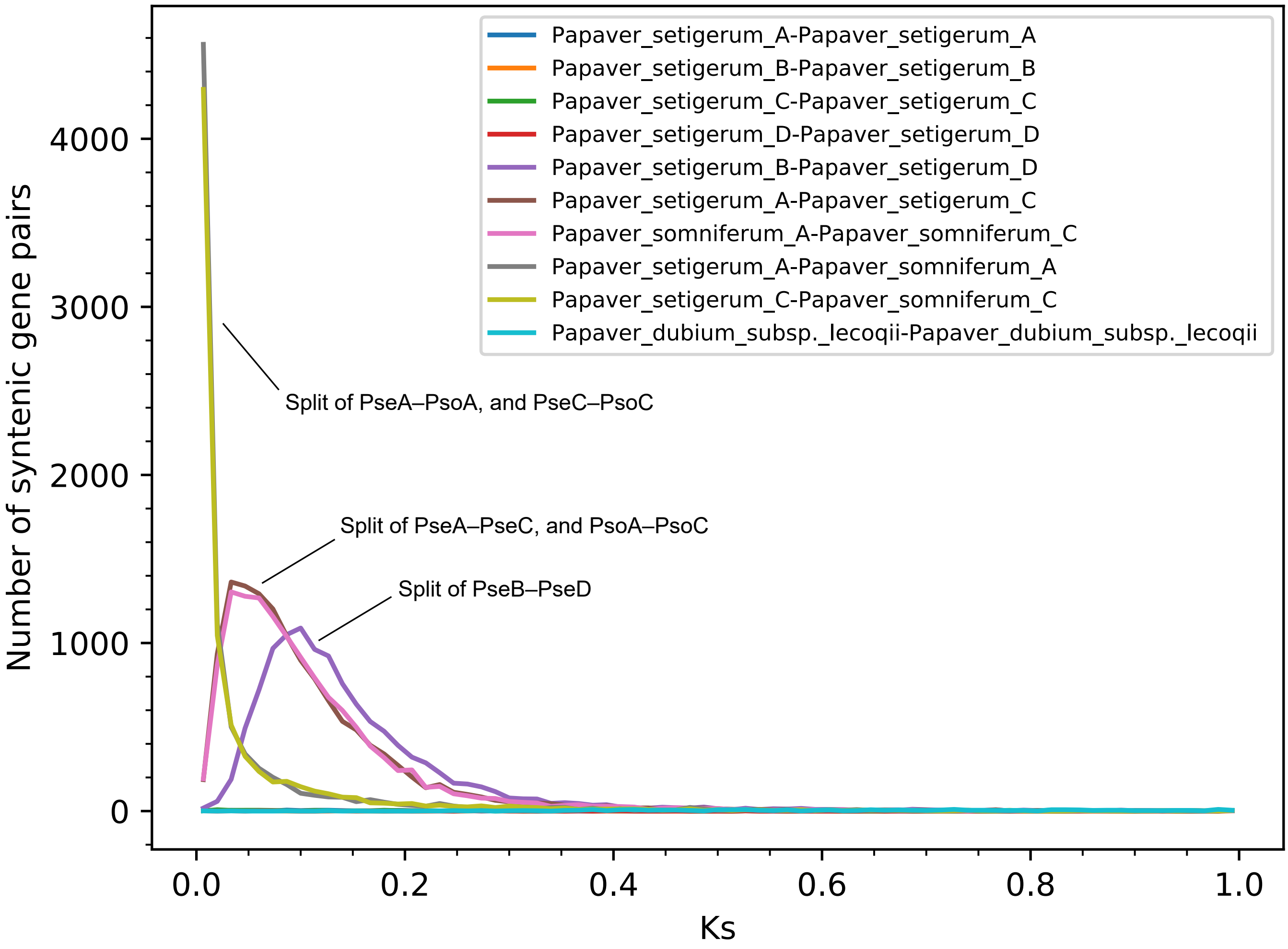

Supplementary Figure 10. ***Ks*** **histogram of paralogs in** ***Papaver dubium.*** The analysis was based on a recently published *P. dubium* subsp. *lecoqii* transcriptome data (TSA: GJOS00000000.1, BioProject: PRJNA770669). Source data are provided as a Source Data file.

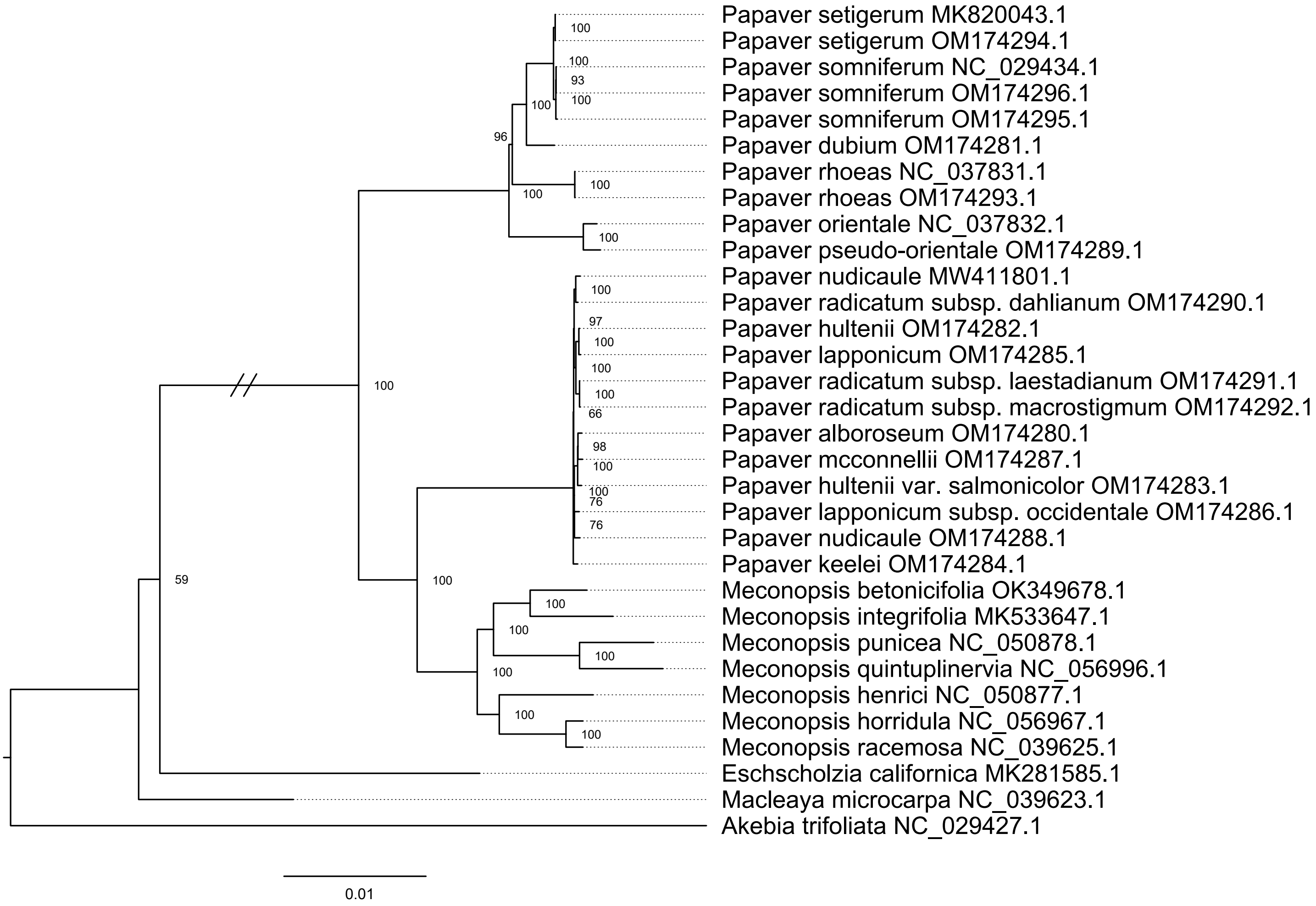

Supplementary Figure 11. Phylogenetic tree based on the complete chloroplast genome sequences of *Papaver* and related taxa. Numbers at the nodes are the bootstrap values calculated by IQ-TREE. Bar, 0.01 substitutions per site. Source data are provided as a Source Data file.

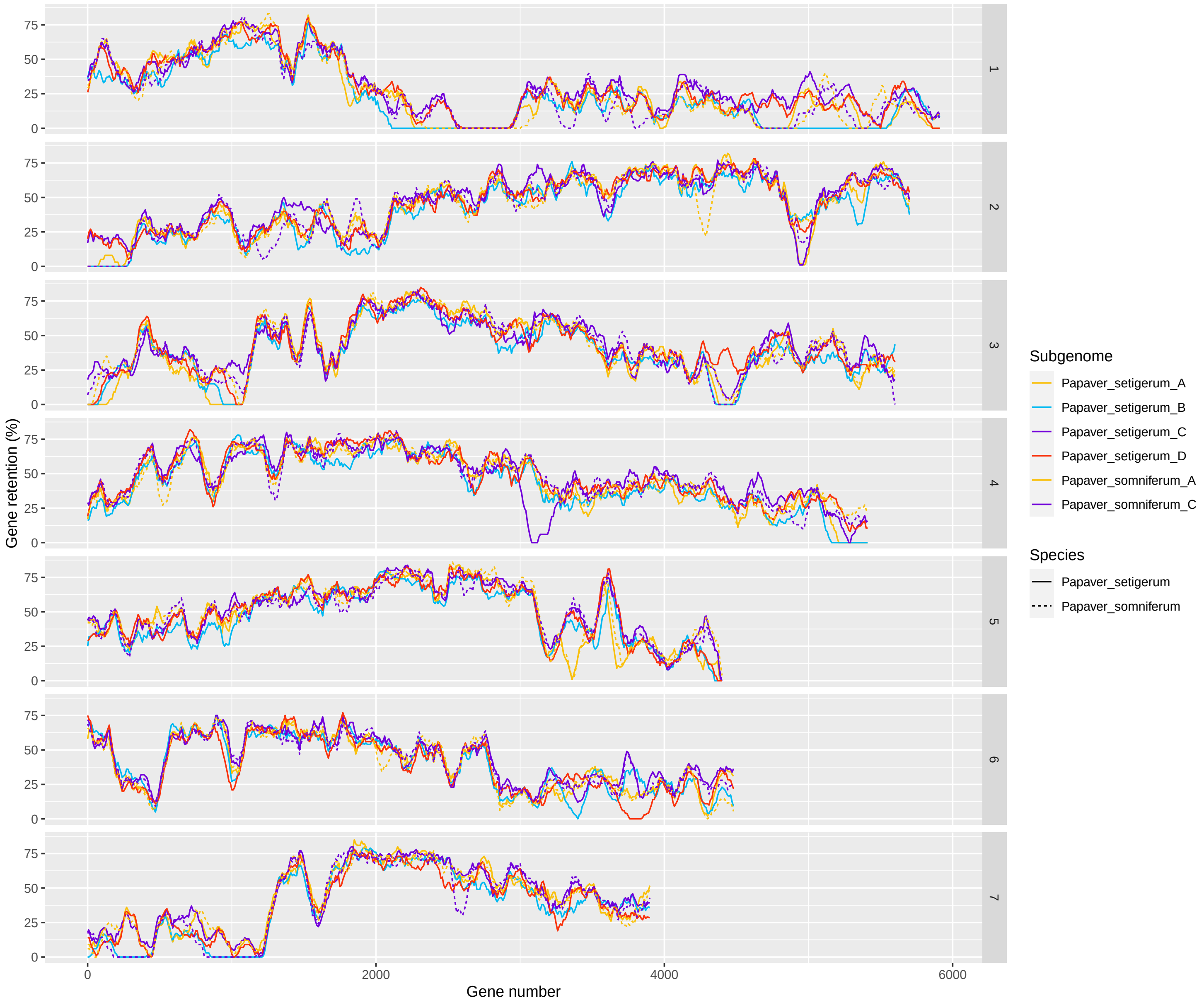

Supplementary Figure 12. Gene retention of subgenomes of *Papaver somniferum* and *Papaver setigerum*. Gene retentions were estimated with a window of 100 genes and a step of 10 genes moving through the reference genome *Papaver rhoeas* (chromosomes 1–7). The retention ratio of syntenic genes in each of the subgenomes for each window was calculated and plotted. Note that SubPhaser failed to assign some chromosomal regions to subgenomes confidently, causing subgenomes to apparently lose all genes in some regions. These regions should not be taken into account. Source data are provided as a Source Data file.

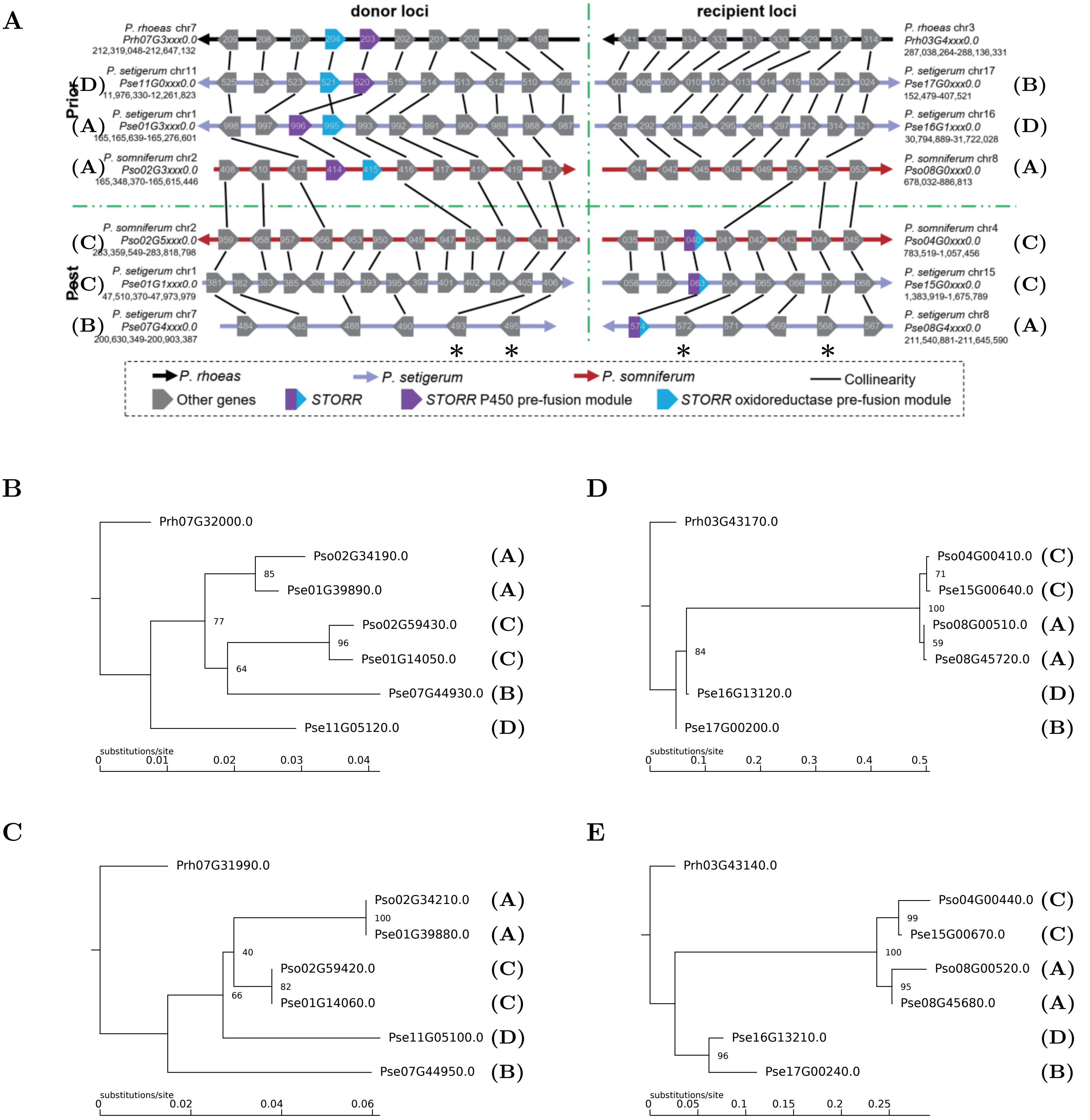

Supplementary Figure 13. Gene phylogenies at the "donor loci" and the "recipient loci" of *STORR*. (**A**) The synteny of the loci was adapted from Supplementary Fig. 25 of Yang et al.[1]. A–D in parentheses indicate subgenome assignments of these loci according to Supplementary Tables 1–2. Asterisks (*) indicate the 1:2:4 homoeologous genes used for reconstructing the following gene trees. (**B–C**) Gene trees at the "donor loci". (**D–E**) Gene trees at the "recipient loci". Numbers at the trees' nodes are bootstrap values calculated using IQ-TREE. Bar, substitutions per site. Letters (i.e. A–D) in parentheses indicate the subgenomic origin. Source data are provided as a Source Data file.

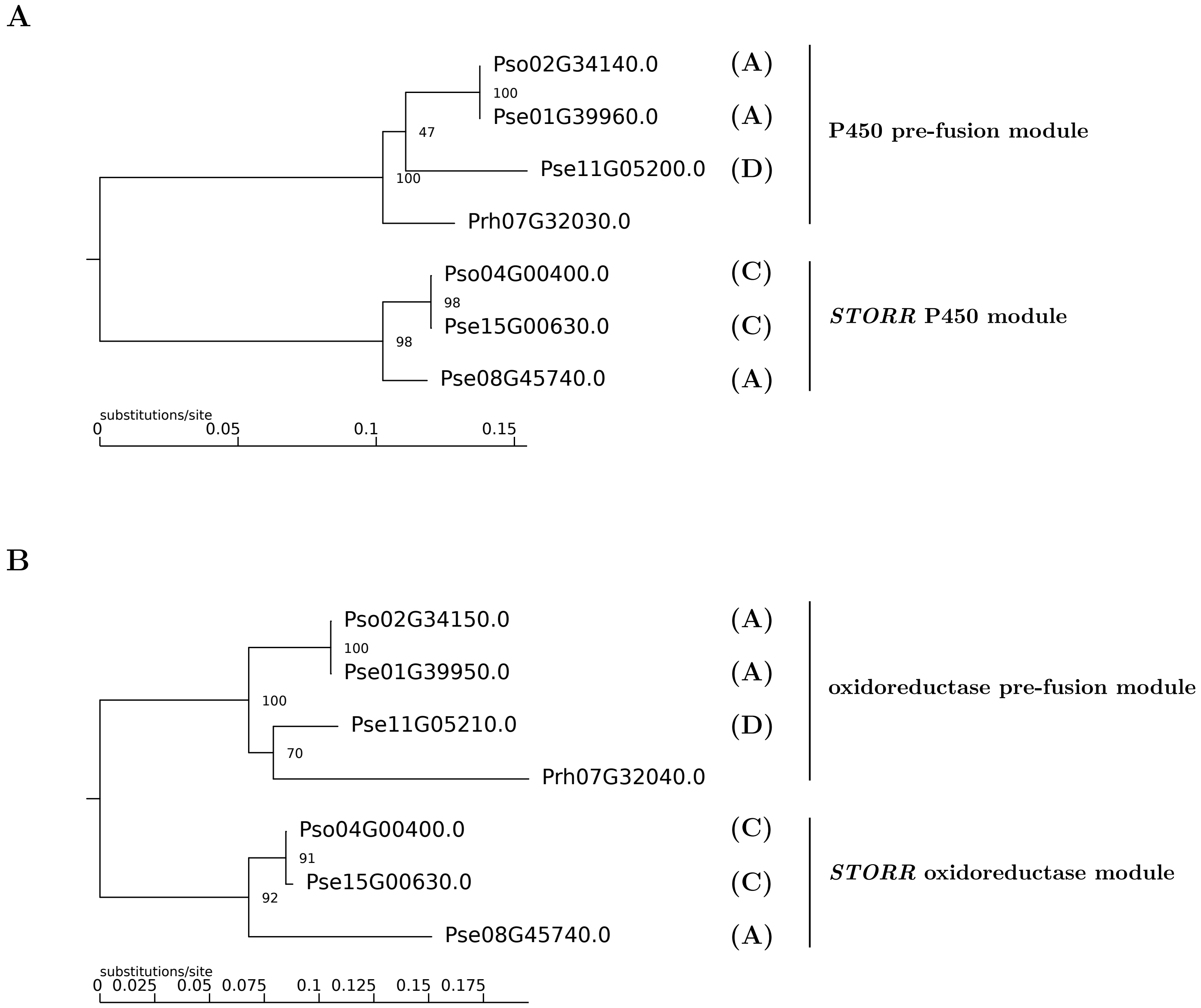

Supplementary Figure 14. Maximum-likelihood gene phylogenies of *STORR* and its pre-fusion modules, P450 (A) and oxidoreductase (B). The gene illustrations can be found in Supplementary Fig. 13. Numbers at the nodes are the bootstrap values calculated using IQ-TREE. Bar, substitutions per site. Letters (i.e. A–D) in parentheses indicate subgenomic locations of genes. The P450 phylogeny (**A**) is similar to the gene tree in Supplementary Fig. 35 of Yang et al.[1] where *CYP82Y1* was used as the outgroup. Source data are provided as a Source Data file.

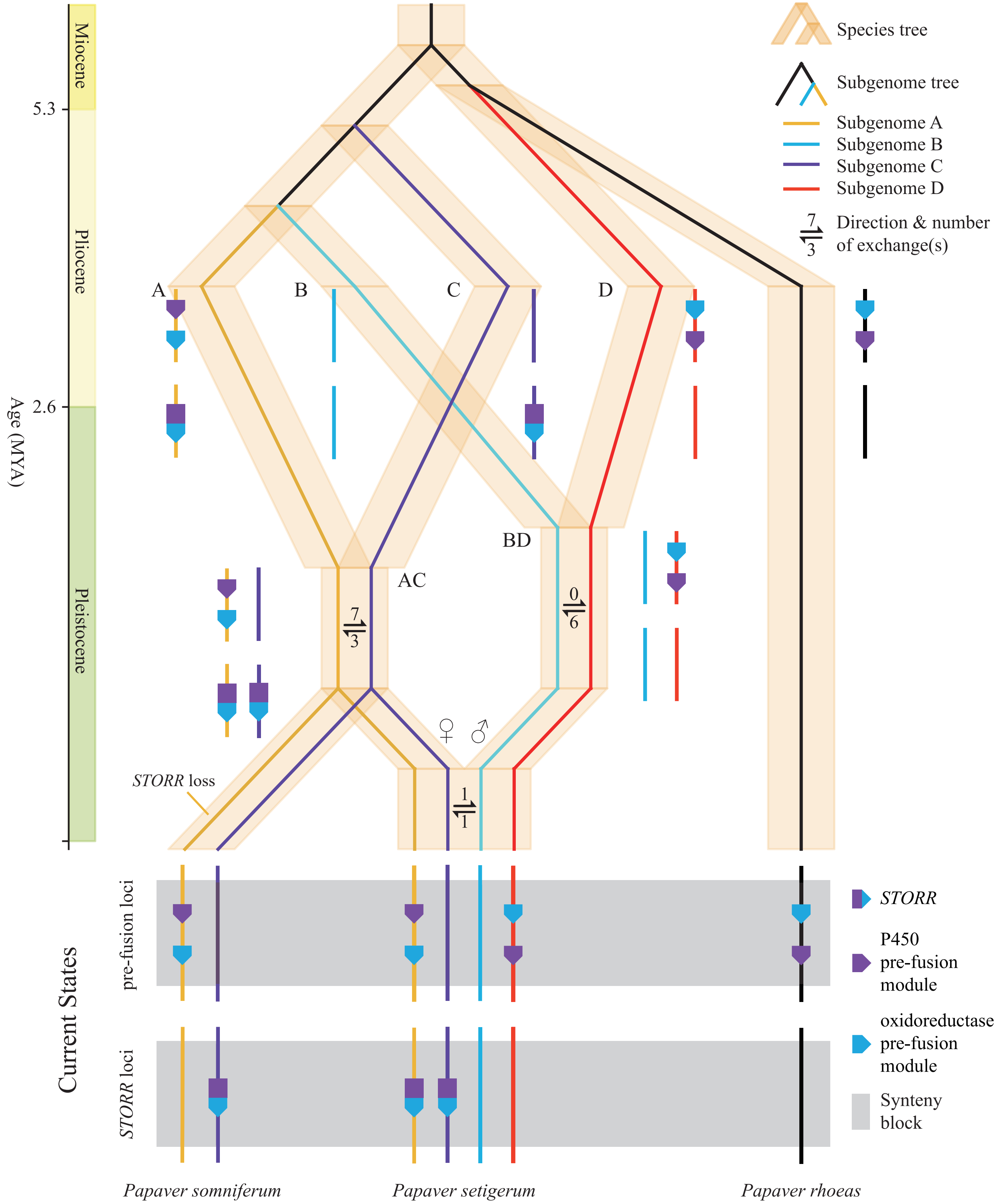

Supplementary Figure 15. An alternative evolutionary scenario of *STORR* and its pre-fusion modules under the reticulate allopolyploidization framework. The bottom panels (current states of *STORR* and its pre-fusion modules) were adapted from Fig. 3a of Yang et al.[1], with the loci being re-colored and re-ordered according to their subgenome assignments in this study. Subgenome assignments of the loci were confirmed by phylogenies of adjacent non-lost 1:2:4 homoeologous genes (Supplementary Fig. 13).

Supplementary Table 1. Subgenomic segments of *Papaver somniferum* identified using SubPhaser. Only segemnts $\geq$ 5 Mb are shown and taken into account.

chromosome	start	end	exchange from	exchange to	number of bins	potential exchange?
chr1	0	6000000	C	C	6	no
chr1	7000000	42000000	C	C	34	no
chr1	43000000	238000000	C	C	194	no
chr1	238000000	253000000	A	C	15	yes
chr6	0	17000000	C	A	17	yes
chr6	22000000	187000000	A	A	165	no
chr7	0	95000000	C	C	95	no
chr7	100000000	235000000	C	C	135	no
chr7	237000000	250000000	C	C	13	no
chr8	0	212000000	A	A	212	no
chr9	0	7000000	A	C	7	yes
chr9	7000000	181000000	C	C	174	no
chr11	0	132000000	A	A	132	no
chr11	133000000	172000000	A	A	39	no
chr4	0	7000000	C	A	7	yes
chr4	8000000	62000000	C	A	54	yes
chr4	62000000	172000000	A	A	110	no
chr5	1000000	185000000	C	C	184	no
chr5	185000000	251000000	A	C	66	yes
chr3	1000000	36000000	A	C	35	yes
chr3	36000000	75000000	C	C	39	no
chr3	81000000	116000000	A	C	35	yes
chr3	116000000	296000000	C	C	180	no
chr10	0	8000000	A	C	8	yes
chr10	8000000	89000000	C	C	81	no
chr10	89000000	117000000	A	C	28	yes
chr10	118000000	136000000	A	C	18	yes
chr10	136000000	165000000	C	C	29	no
chr10	170000000	176000000	C	C	6	no
chr10	177000000	188000000	C	C	11	no
chr10	190000000	197000000	C	C	7	no
chr2	0	26000000	A	A	26	no
chr2	27000000	251000000	A	A	224	no
chr2	253000000	258000000	A	A	5	no
chr2	258000000	310000000	C	A	52	yes
chr2	311000000	328000000	C	A	17	yes
unplaced-scaffold_1	0	20000000	C	C	20	no
unplaced-scaffold_1	20000000	40000000	A	C	20	yes
unplaced-scaffold_2	0	5000000	C	C	5	no
unplaced-scaffold_2	7000000	23000000	C	C	15	no
unplaced-scaffold_2	24000000	32000000	C	C	8	no

Supplementary Table 2. Subgenomic segments of *Papaver setigerum* identified using SubPhaser. Only segemnts $\geq$ 5 Mb are shown and taken into account.

chromosome	start	end	exchange from	exchange to	number of bins	potential exchange?
chr14	4000000	14000000	A	A	8	no
chr14	15000000	177000000	A	A	153	no
chr18	0	10000000	B	B	7	no
chr18	12000000	72000000	B	B	58	no
chr18	73000000	156000000	B	B	70	no
chr19	0	11000000	B	B	9	no
chr19	12000000	37000000	D	B	23	yes
chr19	43000000	150000000	B	B	94	no
chr22	0	111000000	D	D	109	no
chr9	7000000	211000000	C	C	202	no
chr21	0	145000000	D	D	143	no
chr3	0	5000000	C	C	5	no
chr3	6000000	152000000	C	C	140	no
chr3	159000000	266000000	C	C	106	no
chr3	271000000	292000000	A	C	16	yes
chr10	0	67000000	C	C	62	no
chr10	70000000	84000000	A	C	11	yes
chr10	86000000	113000000	A	C	25	yes
chr10	115000000	193000000	C	C	77	no
chr10	193000000	203000000	A	C	9	yes
chr4	0	44000000	C	C	41	no
chr4	45000000	240000000	C	C	192	no
chr4	240000000	254000000	A	C	11	yes
chr6	2000000	27000000	B	B	20	no
chr6	29000000	142000000	B	B	101	no
chr6	156000000	234000000	D	B	78	yes
chr11	0	202000000	D	D	201	no
chr12	0	17000000	C	A	17	yes
chr12	23000000	186000000	A	A	157	no
chr8	0	213000000	A	A	203	no
chr17	0	116000000	B	B	109	no
chr17	122000000	162000000	C	B	37	yes
chr15	0	26000000	C	A	24	yes
chr15	27000000	62000000	C	A	34	yes
chr15	62000000	174000000	A	A	100	no
chr2	3000000	184000000	C	C	176	no
chr2	184000000	219000000	A	C	31	yes
chr2	219000000	262000000	C	C	40	no
chr2	262000000	294000000	A	C	28	yes
chr16	0	169000000	D	D	166	no
chr5	6000000	34000000	B	C	23	yes
chr5	34000000	177000000	C	C	139	no
chr5	177000000	238000000	A	C	55	yes
chr13	0	48000000	D	B	48	yes
chr13	49000000	153000000	B	B	96	no
chr13	155000000	177000000	B	B	17	no
chr20	2000000	124000000	B	B	108	no
chr20	125000000	155000000	B	B	24	no
chr7	0	15000000	D	B	15	yes
chr7	15000000	73000000	B	B	51	no
chr7	73000000	132000000	D	B	59	yes
chr7	136000000	159000000	B	B	19	no
chr7	165000000	211000000	B	B	40	no
chr7	211000000	230000000	D	B	19	yes
chr1	0	75000000	C	A	74	yes
chr1	76000000	241000000	A	A	161	no
chr1	243000000	303000000	A	A	54	no
chr1	304000000	330000000	A	A	26	no

Supplementary Table 3. Split time estimation based on *Ks* values. The time unit is million years ago (MYA).The split times are estimated based on mean, median and peak *Ks* values. The estimations based on median *Ks* values were the most robustness.

SG1	SG2	gene number	mean	median	peak	95% CI	time (mean Ks)	time (median Ks)	time (peak Ks)
Pso-A	Pse-A	8344	0.056	0.011	0.01	0.001-0.432	2.57	0.64	0.7
Pso-C	Pse-C	8119	0.063	0.012	0.01	0.001-0.456	2.90	0.69	0.7
Pse-A	Pse-B	12608	0.115	0.081	0.03	0.017-0.429	5.27	4.69	2.1
Pso-A	Pse-B	12176	0.116	0.083	0.05	0.017-0.434	5.33	4.78	3.5
Pse-A	Pse-C	13571	0.115	0.084	0.05	0.017-0.405	5.26	4.85	3.5
Pso-A	Pse-C	13313	0.119	0.086	0.05	0.017-0.423	5.44	4.97	3.5
Pse-A	Pso-C	13064	0.116	0.085	0.05	0.017-0.420	5.34	4.90	3.5
Pso-A	Pso-C	13440	0.117	0.086	0.05	0.017-0.414	5.38	4.96	3.5
Pse-B	Pse-C	12614	0.127	0.095	0.05	0.021-0.434	5.83	5.47	3.5
Pse-B	Pso-C	12225	0.129	0.096	0.05	0.021-0.455	5.93	5.53	3.5
Pse-A	Pse-D	13100	0.147	0.115	0.09	0.036-0.458	6.76	6.65	6.3
Pso-A	Pse-D	12862	0.148	0.117	0.09	0.035-0.448	6.77	6.74	6.3
Pse-B	Pse-D	11879	0.156	0.123	0.09	0.041-0.483	7.16	7.12	6.3
Pso-C	Pse-D	12990	0.145	0.114	0.07	0.034-0.459	6.65	6.59	4.9
Pse-C	Pse-D	13542	0.141	0.113	0.07	0.034-0.428	6.47	6.49	4.9
Pse-A	Prh	15717	0.166	0.133	0.11	0.044-0.485	7.62	7.66	7.7
Pso-A	Prh	15754	0.169	0.135	0.11	0.044-0.519	7.76	7.76	7.7
Pse-B	Prh	14385	0.175	0.141	0.11	0.048-0.519	8.02	8.11	7.7
Pse-C	Prh	17082	0.166	0.133	0.09	0.043-0.505	7.61	7.65	6.3
Pso-C	Prh	16279	0.167	0.133	0.11	0.043-0.505	7.64	7.64	7.7
Pse-D	Prh	16128	0.157	0.123	0.09	0.040-0.494	7.21	7.09	6.3

Supplementary Table 4. Estimation of time boundaries of the divergence-hybridization period based on insertion ages of subgenome-specific LTRs. The time unit is million years ago (MYA). The lower and upper limits of the periods are estimated based on the 95% percentile-based confidence intervals (CI).

SG1	SG2	mean	median	std	75% CI	95% CI	99% CI	lineage	lower limit (hybridization)	upper limit (divergence)
B-Pse	D-Pse	4.089	3.982	1.474	2.569-5.682	1.170-7.659	0.450-8.644	B-D	0.91	7.3
		3.566	3.674	1.571	1.478-5.190	0.650-6.951	0.314-8.261		(0.65-1.2)	(7.0-7.7)
A-Pse	C-Pse	2.39	2.218	1.359	1.010-3.696	0.442-5.977	0.225-8.184	A-C	0.49	5.8
		2.149	1.933	1.129	1.198-3.052	0.716-5.445	0.308-7.824		(0.26-0.74)	(5.3-6.1)
A-Pse	C-Pso	2.367	2.193	1.367	0.974-3.688	0.426-5.969	0.197-8.184			
		2.025	1.855	1.177	0.970-2.971	0.313-5.315	0.156-7.866			
A-Pso	C-Pse	2.26	2.126	1.422	0.681-3.633	0.259-6.043	0.108-7.922			
		2.173	1.951	1.147	1.218-3.087	0.736-5.598	0.313-7.933			
A-Pso	C-Pso	2.333	2.189	1.416	0.799-3.709	0.274-6.125	0.113-7.990			
		2.153	1.933	1.137	1.217-3.061	0.720-5.528	0.286-7.938			
A-Pse	B-Pse	2.282	2.118	1.339	0.922-3.574	0.403-5.811	0.165-8.016	A-B	0.43	6.2
		2.624	2.193	1.648	0.974-4.602	0.516-6.620	0.247-8.239		(0.26-0.53)	(5.8-6.7)
A-Pso	B-Pse	2.218	2.081	1.372	0.701-3.535	0.255-5.775	0.104-7.782			
		2.669	2.243	1.664	0.990-4.656	0.528-6.722	0.254-8.263			
B-Pse	C-Pse	2.876	2.548	1.737	1.042-4.933	0.542-7.056	0.263-8.436	B-C	0.46	6.3
		2.078	1.879	1.208	0.978-3.103	0.446-5.542	0.211-7.887		(0.31-0.54)	(5.5-7.1)
B-Pse	C-Pso	2.884	2.552	1.744	1.042-4.947	0.544-7.113	0.263-8.432			
		2.056	1.871	1.234	0.918-3.086	0.305-5.589	0.143-7.992			
A-Pse	D-Pse	2.443	2.263	1.418	0.990-3.858	0.422-6.276	0.181-8.189	A-D	0.43	6.5
		2.616	2.18	1.641	0.962-4.553	0.512-6.678	0.259-8.155		(0.27-0.52)	(6.3.-6.7)
A-Pso	D-Pse	2.401	2.247	1.449	0.835-3.836	0.270-6.277	0.116-7.997			
		2.637	2.205	1.649	0.970-4.580	0.521-6.712	0.264-8.192			
C-Pse	D-Pse	2.17	1.933	1.278	1.002-3.318	0.473-5.961	0.238-8.008	C-D	0.45	6.4
		2.669	2.234	1.678	0.966-4.680	0.508-6.786	0.259-8.202		(0.31-0.51)	(5.9-6.9)
C-Pso	D-Pse	2.145	1.924	1.307	0.946-3.292	0.313-5.940	0.147-8.093			
		2.687	2.255	1.691	0.966-4.720	0.508-6.860	0.256-8.205			

Supplementary References

1. Yang, X. et al. Three chromosome-scale *Papaver* genomes reveal punctuated patchwork evolution of the morphinan and noscapine biosynthesis pathway. *Nat. Commun.* **12**, 6030 (2021).

2. Catania, T. et al. A functionally conserved *STORR* gene fusion in *Papaver* species that diverged 16.8 million years ago. *Nat. Commun.* **13**, 3150 (2022).
