# Supplementary material for: Subgenome-aware analyses suggest a reticulate allopolyploidization origin in three Papaver genomes
Source: Nat Commun. 2023 Apr 19;14:2204. doi: 10.1038/s41467-023-37939-2 (PMC10115784; doi:10.1038/s41467-023-37939-2)
Supplement: Supplementary file 2 — Reporting Summary [file 41467_2023_37939_MOESM2_ESM.pdf]

nature portfolio

Corresponding author(s): Wei Zhao, Kai-Hua Jia, Yong-Peng Ma

Last updated by author(s): Mar 30, 2023

Reporting Summary

Nature Portfolio wishes to improve the reproducibility of the work that we publish. This form provides structure for consistency and transparency in reporting. For further information on Nature Portfolio policies, see our Editorial Policies and the Editorial Policy Checklist.

Statistics

For all statistical analyses, confirm that the following items are present in the figure legend, table legend, main text, or Methods section.

n/a	Confirmed	
☐	☒	The exact sample size (*n*) for each experimental group/condition, given as a discrete number and unit of measurement
☒	☐	A statement on whether measurements were taken from distinct samples or whether the same sample was measured repeatedly
☒	☐	The statistical test(s) used AND whether they are one- or two-sided<br>*Only common tests should be described solely by name; describe more complex techniques in the Methods section.*
☒	☐	A description of all covariates tested
☒	☐	A description of any assumptions or corrections, such as tests of normality and adjustment for multiple comparisons
☐	☒	A full description of the statistical parameters including central tendency (e.g. means) or other basic estimates (e.g. regression coefficient) AND variation (e.g. standard deviation) or associated estimates of uncertainty (e.g. confidence intervals)
☒	☐	For null hypothesis testing, the test statistic (e.g. *F*, *t*, *r*) with confidence intervals, effect sizes, degrees of freedom and *P* value noted<br>*Give P values as exact values whenever suitable.*
☒	☐	For Bayesian analysis, information on the choice of priors and Markov chain Monte Carlo settings
☒	☐	For hierarchical and complex designs, identification of the appropriate level for tests and full reporting of outcomes
☒	☐	Estimates of effect sizes (e.g. Cohen's *d*, Pearson's *r*), indicating how they were calculated

Our web collection on statistics for biologists contains articles on many of the points above.

Software and code

Policy information about availability of computer code

Data collection	No software was used for data collection.
Data analysis	OrthoFinder(v2.3.1); MUSCLE(v3.8.425); KaKs_Calculator(v2.0); MAFFT(v7.481); IQ-TREE(v1.6.12); ASTRAL(MP-5.14.5); Trinity(v2.6.6); TransDecoder(v5.2.0); BWA-MEM(v0.7.17); HybPiper(v1.0); SPAdes(v3.13.1); exonerate(v2.2.0); trimAl(v1.2); PAL2NAL(v14); ParaAT(v1.0); MCScanX(v1.0); GetOrganelle(v1.6.2e); OGAP(v1.0); SubPhaser(v1.2); FigTree(v1.3.1); sppIDer(v1.0); bedtools(v2.27.1).

For manuscripts utilizing custom algorithms or software that are central to the research but not yet described in published literature, software must be made available to editors and reviewers. We strongly encourage code deposition in a community repository (e.g. GitHub). See the Nature Portfolio guidelines for submitting code & software for further information.

Data

Policy information about availability of data

All manuscripts must include a data availability statement. This statement should provide the following information, where applicable:

- Accession codes, unique identifiers, or web links for publicly available datasets
- A description of any restrictions on data availability
- For clinical datasets or third party data, please ensure that the statement adheres to our policy

The chloroplast genome sequences assembled in this study have been deposited in the GenBank database under accession codes OM174280 [https://www.ncbi.nlm.nih.gov/nuccore/OM174280]–OM174296 [https://www.ncbi.nlm.nih.gov/nuccore/OM174296]. Assemblies of transcriptome and genome skimming

data of Papaver generated in this study are available at figshare (https://doi.org/10.6084/m9.figshare.20323995.v1). Genome assemblies of P. rhoeas, P. somniferum and P. setigerum were download from National Genomics Data Center (NGDC) Genome Warehouse (GWH) database under BioProject accession PRJCA004217 [https://ngdc.cncb.ac.cn/bioproject/browse/PRJCA004217]. Gene annotations of P. rhoeas, P. somniferum and P. setigerum were downloaded from GitHub (https://github.com/xjtu-omics/Papaver-Genomics/). Raw genome sequencing reads of P. rhoeas, P. somniferum and P. setigerum were downloaded from the NCBI Sequence Read Archive (SRA) database under BioProject accession PRJNA720042 [https://www.ncbi.nlm.nih.gov/sra/?term=PRJNA720042]. Gene annotations of Macleaya cordata, Kingdonia uniflora, Tetracentron sinense, Coptis chinensis, and Prunus persica were downloaded from the NCBI GenBank/RefSeq databases under accessions GCA_002174775.1 [https://www.ncbi.nlm.nih.gov/assembly/GCA_002174775.1/], GCA_014058105.1 [https://www.ncbi.nlm.nih.gov/assembly/GCA_014058105.1/], GCA_015143295.1 [https://www.ncbi.nlm.nih.gov/assembly/GCA_015143295.1/], GCA_015680905.1 [https://www.ncbi.nlm.nih.gov/assembly/GCA_015680905.1/] and GCF_000346465.2 [https://www.ncbi.nlm.nih.gov/assembly/GCF_000346465.2/], respectively. Gene annotations of Vitis vinifera v2.1 (https://phytozome-next.jgi.doe.gov/info/Vvinifera_v2_1) and Aquilegia coerulea v3.1 (https://phytozome-next.jgi.doe.gov/info/Acoerulea_v3_1) were taken from the Phytozome database. Gene annotations of Macadamia integrifolia were from the NGDC GWH database under accession GWHBAUK00000000.1 [https://ngdc.cncb.ac.cn/gwh/Assembly/23196/show]. Gene annotations of Amborella trichopoda v6.1 were from the CoGe database (https://genomevolution.org/coge/GenomeInfo.pl?gid=50948). Gene annotations of Trochodendron aralioides were taken from the GigaDB database (http://gigadb.org/dataset/100657). Gene annotation of Coffea canephora were downloaded from the Coffee Genome Hub (http://coffee-genome.org). Gene annotations of Nelumbo nucifera China Antique v2.0 were downloaded from the Nelumbo Genome Database (http://nelumbo.biocloud.net). Gene annotations of Eschscholzia californica v1.0 were from the Eschscholzia Genome DataBase (http://eschscholzia.kazusa.or.jp). Gene annotations of Aquilegia oxysepala (https://doi.org/10.1038/s41438-020-0328-y) and Akebia trifoliata (https://doi.org/10.1038/s41438-020-00458-y) were obtained from corresponding authors. Raw genome skimming sequencing data of 16 Papaver species were downloaded from the NCBI SRA database under BioProject accession PRJEB43865 [https://www.ncbi.nlm.nih.gov/bioproject/?term=PRJEB43865]. Raw transcriptome sequencing data of P. bracteatum were downloaded from the NCBI SRA database under BioProject accession PRJEB21674 [https://www.ncbi.nlm.nih.gov/bioproject/?term=PRJEB21674]. A transcriptome shotgun assembly of P. dubium subsp. lecoqii was downloaded from the NCBI GenBank database under the accession GJOS00000000.1 [https://www.ncbi.nlm.nih.gov/nuccore/GJOS00000000.1]. Chloroplast genome sequences of Papaver and related taxa were downloaded from the NCBI GenBank/RefSeq databases with accessions MK820043.1 [https://www.ncbi.nlm.nih.gov/nuccore/MK820043.1], NC_029434.1 [https://www.ncbi.nlm.nih.gov/nuccore/NC_029434.1], NC_037831.1 [https://www.ncbi.nlm.nih.gov/nuccore/NC_037831.1], NC_037832.1 [https://www.ncbi.nlm.nih.gov/nuccore/NC_037832.1], MW411801.1 [https://www.ncbi.nlm.nih.gov/nuccore/MW411801.1], OK349678.1 [https://www.ncbi.nlm.nih.gov/nuccore/OK349678.1], MK533647.1 [https://www.ncbi.nlm.nih.gov/nuccore/MK533647.1], NC_050878.1 [https://www.ncbi.nlm.nih.gov/nuccore/NC_050878.1], NC_056996.1 [https://www.ncbi.nlm.nih.gov/nuccore/NC_056996.1], NC_050877.1 [https://www.ncbi.nlm.nih.gov/nuccore/NC_050877.1], NC_056967.1 [https://www.ncbi.nlm.nih.gov/nuccore/NC_056967.1], NC_039625.1 [https://www.ncbi.nlm.nih.gov/nuccore/NC_039625.1], MK281585.1 [https://www.ncbi.nlm.nih.gov/nuccore/MK281585.1], NC_039623.1 [https://www.ncbi.nlm.nih.gov/nuccore/NC_039623.1], and NC_029427.1 [https://www.ncbi.nlm.nih.gov/nuccore/NC_029427.1]. Source data are provided with this paper.

Human research participants

Policy information about studies involving human research participants and Sex and Gender in Research.

Reporting on sex and gender	N/A
Population characteristics	N/A
Recruitment	N/A
Ethics oversight	N/A

Note that full information on the approval of the study protocol must also be provided in the manuscript.

Field-specific reporting

Please select the one below that is the best fit for your research. If you are not sure, read the appropriate sections before making your selection.

☒ Life sciences ☐ Behavioural & social sciences ☐ Ecological, evolutionary & environmental sciences

For a reference copy of the document with all sections, see nature.com/documents/nr-reporting-summary-flat.pdf

Life sciences study design

All studies must disclose on these points even when the disclosure is negative.

Sample size	The data used in this manuscript are all downloaded from the Internet. Therefore, sample size is not applicable to the study.
Data exclusions	The filtering process did not result in any sample exclusions, data filtering was performed to read data.
Replication	Replication was not applicable in this study.
Randomization	Randomization was not applicable in this study.
Blinding	Blinding test was not performed as it was not relevant to this study.

Reporting for specific materials, systems and methods

We require information from authors about some types of materials, experimental systems and methods used in many studies. Here, indicate whether each material, system or method listed is relevant to your study. If you are not sure if a list item applies to your research, read the appropriate section before selecting a response.

Materials & experimental systems

n/a	Involved in the study	
☒	☐	Antibodies
☒	☐	Eukaryotic cell lines
☒	☐	Palaeontology and archaeology
☒	☐	Animals and other organisms
☒	☐	Clinical data
☒	☐	Dual use research of concern

Methods

n/a	Involved in the study	
☒	☐	ChIP-seq
☒	☐	Flow cytometry
☒	☐	MRI-based neuroimaging
